# Ketogenic Essential Amino Acids Modulate Lipid Synthetic Pathways and Prevent Hepatic Steatosis in Mice

**DOI:** 10.1371/journal.pone.0012057

**Published:** 2010-08-10

**Authors:** Yasushi Noguchi, Natsumi Nishikata, Nahoko Shikata, Yoshiko Kimura, Jose O. Aleman, Jamey D. Young, Naoto Koyama, Joanne K. Kelleher, Michio Takahashi, Gregory Stephanopoulos

**Affiliations:** 1 Department of Chemical Engineering, Massachusetts Institute of Technology, Cambridge, Massachusetts, United States of America; 2 Research Institute for Health Fundamentals, Ajinomoto Co. Inc., Kawasaki, Kanagawa, Japan; 3 Shriners Burn Hospital, Massachusetts General Hospital, Boston, Massachusetts, United States of America; Institute of Preventive Medicine, Denmark

## Abstract

**Background:**

Although dietary ketogenic essential amino acid (KAA) content modifies accumulation of hepatic lipids, the molecular interactions between KAAs and lipid metabolism are yet to be fully elucidated.

**Methodology/Principal Findings:**

We designed a diet with a high ratio (E/N) of essential amino acids (EAAs) to non-EAAs by partially replacing dietary protein with 5 major free KAAs (Leu, Ile, Val, Lys and Thr) without altering carbohydrate and fat content. This high-KAA diet was assessed for its preventive effects on diet-induced hepatic steatosis and whole-animal insulin resistance. C57B6 mice were fed with a high-fat diet, and hyperinsulinemic *ob/ob* mice were fed with a high-fat or high-sucrose diet. The high-KAA diet improved hepatic steatosis with decreased *de novo* lipogensis (DNL) fluxes as well as reduced expressions of lipogenic genes. In C57B6 mice, the high-KAA diet lowered postprandial insulin secretion and improved glucose tolerance, in association with restored expression of muscle insulin signaling proteins repressed by the high-fat diet. Lipotoxic metabolites and their synthetic fluxes were also evaluated with reference to insulin resistance. The high-KAA diet lowered muscle and liver ceramides, both by reducing dietary lipid incorporation into muscular ceramides and preventing incorporation of DNL-derived fatty acids into hepatic ceramides.

**Conclusion:**

Our results indicate that dietary KAA intake improves hepatic steatosis and insulin resistance by modulating lipid synthetic pathways.

## Introduction

Non-alcoholic fatty liver disease (NAFLD) caused by persistent hepatic steatosis affects up to one-third of the US population [1], [2]. Because NAFLD is associated with hepatic insulin resistance and can further progress to non-alcoholic steatohepatitis (NASH), there is a critical need to elucidate the molecular pathogenesis of NAFLD so that nutritional strategies can be developed for its prevention and treatment [1], [3]. In particular, elevated concentrations of “lipotoxic lipids” such as diacylglycerols and ceramides have been recognized as factors contributing to impaired insulin signaling in non-adipose tissues [4], [5]. Understanding how to effectively modulate the levels of these lipid intermediates through dietary interventions is a key step toward controlling NAFLD and related disorders.

Previous studies have shown that dietary withdrawal of the ketogenic amino acid (KAA) lysine or threonine induces severe hepatic steatosis in rodents [6], [7]. Furthermore, a role for the amino acid deprivation sensor GCN2 in regulating hepatic lipid homeostasis has been recently revealed [8]. Certain KAAs, especially leucine, are reported to modulate insulin signaling via the mammalian target of rapamycin complex 1 (mTORC1) and the downstream ribosomal protein S6 kinase 1 (S6K1) [9], [10]. Activation of mTORC1 by nutritional overloading is believed to induce insulin resistance in obese subjects [11]. In fact, continuous infusion of amino acids has been shown to induce insulin resistance in human muscle through activation of the mTOR pathway [12]. More recently, the combination of dietary branched-chain amino acids (BCAAs) and fat over-intake was shown to induce insulin resistance in rats [13] .

In contrast to those studies exhibiting detrimental effects of amino acids on NAFLD and insulin signaling, several clinical trials and animal experiments have demonstrated that KAA supplementation can have beneficial effects on insulin sensitivity and/or obesity. For instance, leucine feeding in mice attenuated high-fat-induced obesity, hyperglycemia and hypercholesterolemia [14], and an orally administered KAA mixture of leucine, isoleucine, valine, threonine and lysine improved insulin sensitivity in elderly patients with type-2 diabetes [15]. Furthermore, increased availability of BCAAs in knockout mice harboring a deletion of mitochondrial BCAA transaminase (BCATm) preserved muscle insulin sensitivity in response to long-term high-fat feeding [16]. Thus, the role of essential amino acids (EAAs), and KAAs in particular, in the etiology of insulin resistance and hepatic steatosis remains controversial.

In the present study, we designed a novel diet with an elevated ratio of EAA to non-EAA (high-E/N diet) and combined it with either high-fat or high-sucrose feeding. A substantial fraction of dietary protein in the high-E/N diet was replaced with a mixture of 5 free KAAs (leucine, isoleucine, valine, lysine and threonine) without altering dietary carbohydrate and fat content. We demonstrate that dietary KAA fortification prevented hepatic steatosis in mouse models of diet-induced obesity (DIO). Measurement of lipid species and lipogenic fluxes provided further insight into the underlying preventive mechanism.

## Results

### Manipulation of dietary E/N ratio by partial protein replacement with free KAA

**Figure 1:**
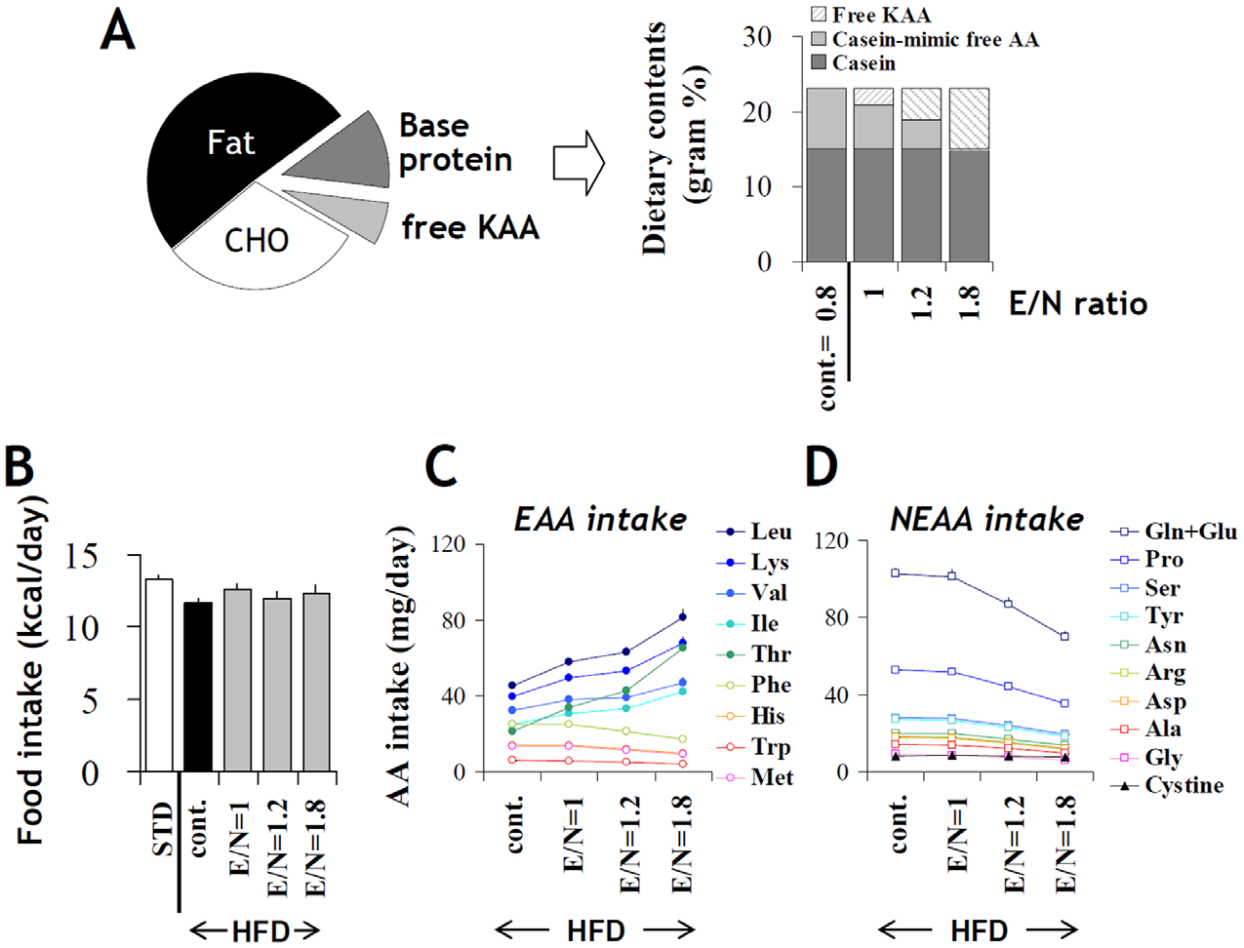
Summary of dietary ketogenic amino acid manipulation. (A) High ketogenic amino acid (KAA) diets were prepared by replacing dietary protein with a free KAA mixture of leucine, lysine, isoleucine, valine and threonine (for 1.8 E/N-ratio diet) or KAA plus casein-mimic free amino acid mixture (for 1 and 1.2 E/N-ratio diets). (B) Food intake by C57B6 mice was calculated on the basis of mean energy consumption (kcal/day) throughout an 8-week experimental period. Daily intake (mean+/−SEM, n = 9 for each group) of essential amino acids (EAA) (C) and non-EAA (NEAA) (D) in mice fed a high-fat diet with varied ratios of E/N (Table S1) were calculated based on daily food intake and amino acid composition of each diet. The five major KAA supplemented to the high E/N diets are distinguished by closed symbols.

In the present study, the amino acid composition of a standard low-fat diet (STD), high-fat diet (HFD) or high-sucrose diet (HSD) was manipulated by replacing a fraction of dietary proteins with a mixture of free amino acids. For instance, two different types of the high-fat diet were prepared. The first one was a control diet containing 15% of basal casein and 8% of a free amino acid mixture, which replicated the original casein amino acid composition (“casein-mimic free AA”). The other was a high-KAA diet containing also 15% of basal casein and 8% of a selected KAA mixture in place of the casein-mimic free AAs (Figure 1A). This increased dietary E/N ratio from 0.8 (control) to 1.8 depending on the level of KAA replacement. Five major KAAs most commonly found in dietary proteins—leucine, isoleucine, valine, lysine and threonine—were included in the KAA mixture. The nutrient and energy compositions of the diets used in the study are compared in Supplemental Table S1. The total amount of amino acids was normalized among diets by adding varied amounts of the casein-mimic free AA mixture, if necessary.

When fed to C57B6 mice over an 8-week experimental period, caloric intake was not significantly different across the various diets (Figure 1B). Therefore, high-E/N diet ingestion resulted in considerably higher amounts of KAA intake (BCAA, lysine and threonine increased between 28% and 85% in comparison to control) and moderately decreased intake of other amino acids (between 2% and 32% in comparison to control) (Figures 1C, D).

### Effect of high-KAA diet on physiologic alterations induced by high-fat feeding

**Figure 2:**
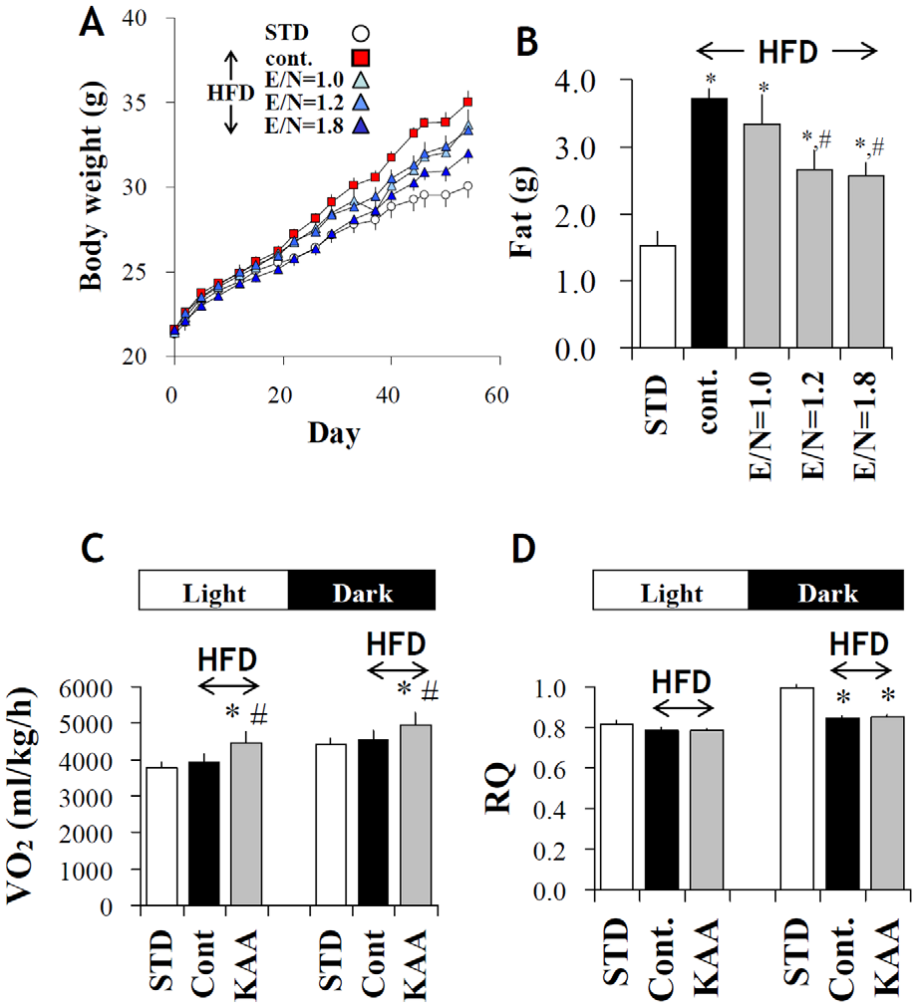
Effects of high KAA diets on high-fat induced alterations. Growth curves (A), fat (subcutaneous, epididymal and perinephric) weights (B), oxygen consumption (C), and respiratory quotient (D) of C57B6 mice fed for 8 weeks with varying E/N ratios due to KAA fortification (mean+/−SEM, n = 9 for each group). For oxygen consumption and respiratory quotient, mean values were obtained over 3 days following a week of acclimatization to the metabolic chamber, and only the results from the highest KAA diet (E/N = 1.8) are separately presented in the light and dark phase. *: p<0.05 for all the HFD control groups as compared to STD group; #: p<0.05 for all the high KAA (high E/N) groups as compared to HFD control.

We confirmed in a preliminary experiment that increases in dietary protein (casein) levels from 15 to 25% had only slight effects on physiologic alterations induced by high-fat feeding in C57B6 mice, except that plasma cholesterol level increased significantly on a 25% casein diet (Figure S1). In contrast to this “high-protein diet”, the high-KAA diets (E/N>1) suppressed many of the physiologic alterations induced by high-fat feeding in a E/N-dependent manner. Body weight gain due to high-fat feeding was significantly reduced with increasing E/N ratio (Figure 2A). At the end of an 8-week experimental period, fat weight decreased by 31% in the E/N = 1.8 group as compared with the control HFD group (Figure 2B). Oxygen consumption in the light and dark phase increased by 13 and 9%, respectively (Figure 2C). On the other hand, although respiratory quotient (RQ) decreased to 0.8 in the dark phase in HFD groups, it was not affected by KAA fortification (Figure 2D). Since high-protein diets are known to increase kidney weight in rodents, we determined whether KAA fortification would elicit a similar response. However, no significant increase was observed, suggesting that the high-E/N diets used in this study do not lead to protein overloading effects (data not shown).

Table 1 illustrates postprandial variables at the end of the 8-week experimental period. High-fat feeding of wild-type mice for 8 weeks is sufficient to induce hyperinsulinemia but not apparent hyperglycemia. The high-KAA diet significantly reduced postprandial insulin as well as plasma cholesterol, while increasing β-hydroxybutyrate. In addition, the high-KAA diet significantly reduced leptin, due to the reduction of fat weight. There were no clear differences in plasma glucose, FFA, IL-6, TNF-α, and resistin among 4 high-fat groups irrespective of KAA fortification. The high-KAA diet had no effect on most plasma amino acid levels except for threonine, which increased approximately 2-fold at E/N = 1.8 relative to E/N = 0.8 (Table S4).

**Table 1:** Plasma metabolic parameters in C57B6 mice fed HFD with or without high E/N manipulation by KAA.

	STD	HFD	HFD+KAA		
E/N ratio	0.8	0.8	1.0	1.2	1.8
	(n = 9)	(n = 9)	(n = 9)	(n = 9)	(n = 9)
Glucose (mmol/l)	11.8±0.6	14.1±0.4^*^	13.6±0.5^*^	12.8±0.6	13.7±0.6^*^
Triglyceride (mg/dl)	72±6	70±3	65±4	77±10	64±3
FFA (mmol/l)	0.48±0.03	0.38±0.03^*^	0.35±0.03^*^	0.37±0.03^*^	0.38±0.03^*^
Cholesterol (mg/dl)	117±5	158±6^*^	145±7^*^	132±5^#^	128±5^#^
Acetoacetate (µmol/l)	144±4	131±3^*^	133±3^*^	136±6	137±4
β-OH-butyrate (µmol/l)	203±31	185±20	167±16	192±32	249±33^#^
Insulin (ng/ml)	0.8±0.1	3.7±0.5^*^	2.7±0.5^*^	2.1±0.3^*,#^	1.8±0.3^*,#^
Leptin (ng/ml)	4.3±0.4	40.5±9.0^*^	31.5±7.0^*^	14.2±3.6^*,#^	13.3±3.4^*,#^
IL-6(pg/ml)	N.A.	4.6±0.4	4.7±0.2	4.3±0.7	4.5±0.4
TNFα(ng/ml)	N.A.	11.6±0.1	11.6±0.0	11.6±0.0	11.6±0.3
Resistin (ng/ml)	N.A.	6.3±0.5	6.9±0.6	5.0±0.6	5.0±0.6

Data represent mean +/− SE. *, p<0.05 for all high E/N groups compared with STD group (E/N = 0.8); #, p<0.05 for high E/N groups with HFD control. N.A., not analyzed.

### Effects of high-KAA diet on hepatic steatosis and its metabolic consequences

**Figure 3:**
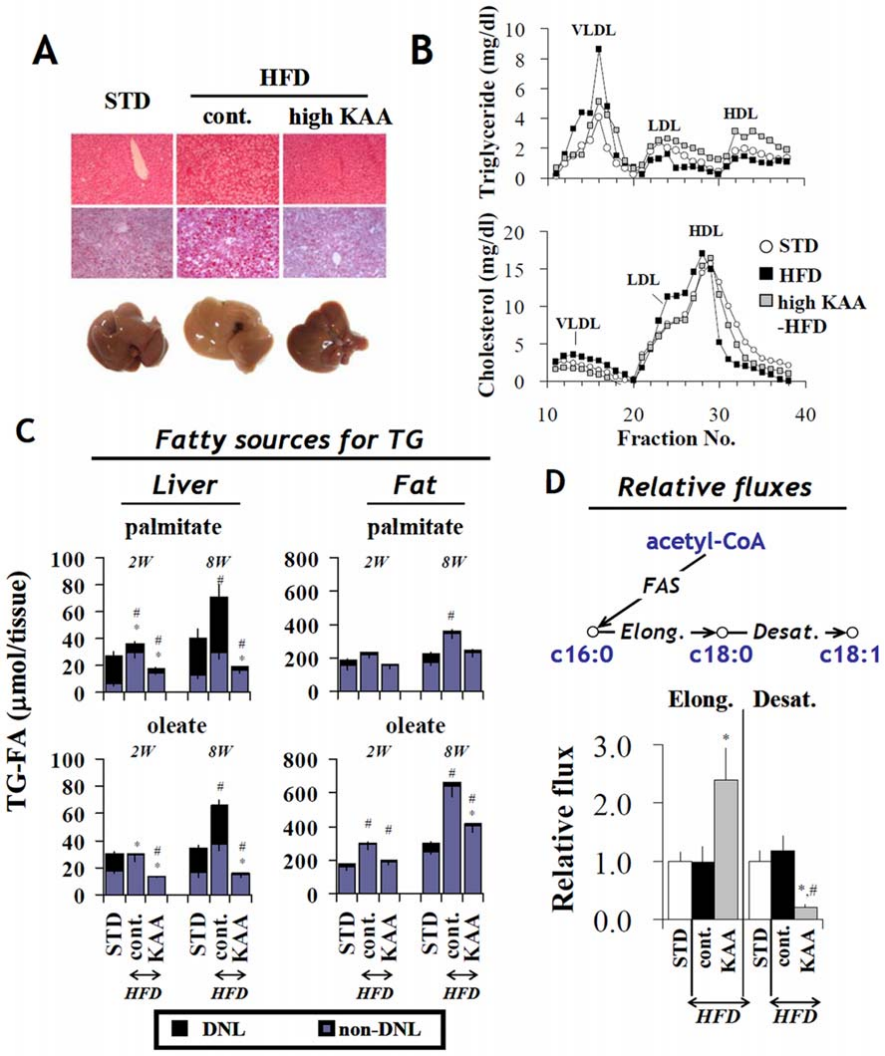
Prevention of high-fat induced hepatic steatosis by high-KAA diet. Samples were obtained from C57B6 mice fed for 2 or 8 weeks with STD, HFD or high-KAA HFD (E/N = 1.8). (A) Liver histology of hematoxylin-eosin staining (top) and oil red O staining (middle), and macroscopic appearances (bottom). (B) FPLC analyses of frozen plasma triglycerides (top) and cholesterol (bottom). Fractions 15–19: very low density lipoprotein (VLDL) and chylomicrons; fractions 20–26: intermediate density lipoproteins, low density lipoprotein (LDL), and large high density lipoprotein; fractions 27–33: high density lipoprotein (HDL). (C, D) Metabolic fluxes of *de novo* lipogenesis (DNL) pathways were analyzed by identifying fatty sources for hepatic triglycerides (C) and estimating the *in vivo* relative contributions of fatty acid synthase (FAS), elongase and desaturase fluxes using deuterated water labeling and mass isotopomer distribution analysis (D) (see Materials and Methods). The contribution of DNL to total triglyceride-fatty acids (TG-FA) in the liver (C, left) or epididymal fat tissue (C, right) was separately estimated for palmitate (c16:0) and oleate (c18:1). Black and blue bars represent DNL- and non-DNL sources, respectively. The contributions of elongase and desaturase (D) were assessed by DNL_c18:0_/DNL_c16:0_ and DNL_c18:1_/DNL_c18:0_, respectively. All values are expressed as mean+/−SEM (n = 6). In panel C, p<0.05 of DNL-derived FA and total FA compared to STD are indicated as * and #, respectively. In panel D, *: p<0.05 for all high-fat groups as compared to STD group; #: p<0.05 for the high-KAA group as compared to HFD control.

Hepatic lipid content and histology were compared in C57B6 mice between groups fed with either a standard diet (STD), high-fat diet (cont), or KAA-fortified high-fat diet (high E/N) for 8 weeks. The high-KAA diet (E/N = 1.8) noticeably improved hepatic steatosis (Figure 3A) and significantly reduced liver weight coinciding with reductions in hepatic triglyceride and cholesterol content of 72% and 59%, respectively (Table 2). The high-KAA diet also normalized plasma VLDL-triglyceride and LDL-cholesterol elevations caused by HFD feeding (Figure 3B).

**Table 2:** Hepatic lipid contents in C57B6 mice fed HFD with or without high E/N manipulation by KAA.

	STD	HFD	
E/N ratio	0.8	0.8	1.8
	(n = 9)	(n = 9)	(n = 9)
Liver (g)	1.6±0.1	1.9±0.1^*^	1.2±0.0^#^
Triglyceride (mg/g)	106±29	209±17^*^	60±12^#^
Cholesterol (mg/g)	9.0±0.3	13.5±0.5^*^	5.5±0.5^#^

Data represent mean +/− SE. C57B6 mice were housed with indicated diets for 8 weeks. Liver lipids were determined as described in Materials and Methods. *, p<0.05 for high E/N groups compared with STD group (E/N = 0.8); #, p<0.05 for high E/N group (E/N = 1.8) with HFD control (E/N = 0.8).

To further understand the antagonizing effects of the high-KAA diet on hepatic steatosis, metabolic flux analysis was applied to quantify changes in *de novo* lipogenesis (DNL) pathways. Figure 3C summarizes different fatty acid sources contributing to hepatic and adipose triglyceride (TG) in C57B6 mice at 2 and 8 weeks. The contribution of DNL to TG-palmitate (c16:0), TG-stearate (c18:0) and TG-oleate (c18:1) in fat tissue was generally small and was further decreased with HFD feeding. In contrast, the contribution of DNL to hepatic TG-palmitate and TG-oleate was substantial at 8 weeks and was further increased by HFD feeding. However, hepatic incorporation of DNL-derived fatty acids into TG was completely suppressed by the high-KAA diet. Relative hepatic fluxes clearly indicate a dissociation of elongase and desaturase pathways, with simultaneous elevation of elongase flux and suppression of desaturase flux (Figure 3D).

**Figure 4:**
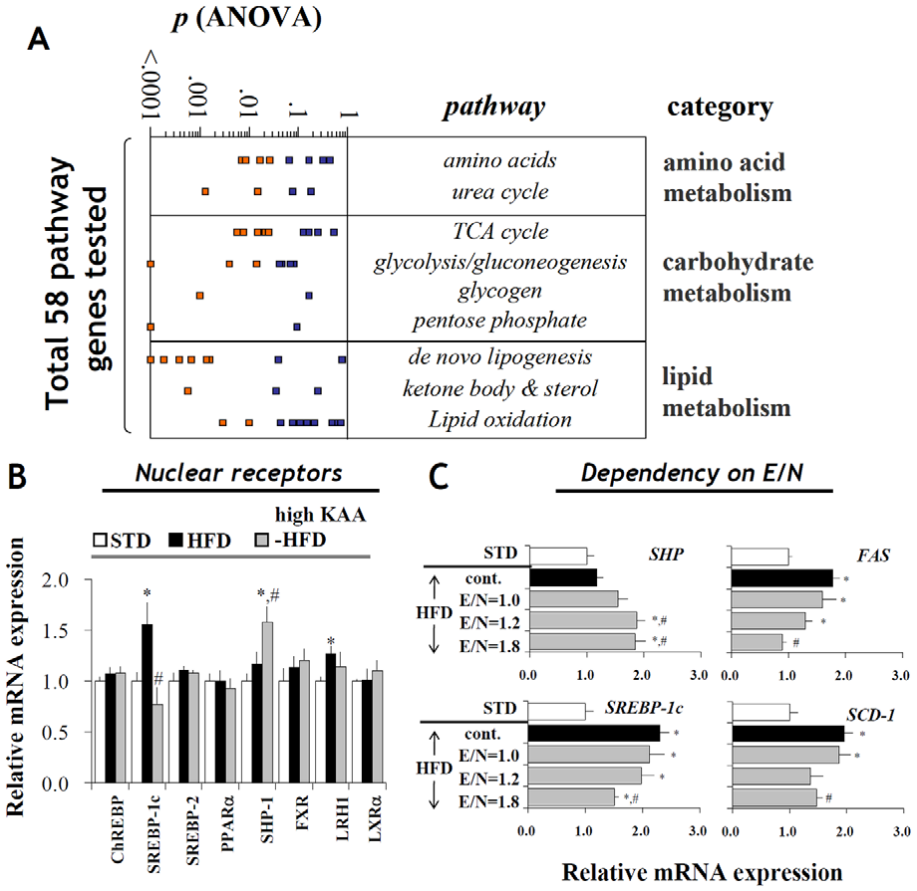
Changes in expression profiles of hepatic metabolic genes and regulators under different dietary conditions. Samples were obtained from C57B6 mice (n = 9 for each group) fed for 8 weeks with STD, HFD or high-KAA (high E/N) HFD. (A) Diet-dependent changes in expressions of 70 hepatic genes (Table S6) involved in different metabolic pathways were assessed by ANOVA probability. Orange squares represent genes having significantly different expression levels (p<0.05) among 3 different diets (HFD, STD, and HFD plus KAA (E/N = 1.8)). Relative gene expression levels (STD = 1, normalized with 18S ribosomal RNA) of representative transcription factors and nuclear receptors (B), and fatty acid synthase (FAS), stearoyl-CoA desaturase 1 (SCD1), SREBP-1c and SHP under different dietary conditions (C). All values are expressed as mean+/−SEM. *: p<0.05 for all high-fat groups as compared to STD group; #: p<0.05 for the high-KAA (high-E/N) groups as compared to HFD control.

To confirm these findings, liver expression profiles of 70 genes involved in amino acid, carbohydrate and lipid metabolism were measured in response to HFD feeding with or without KAA supplementation. Significance level of each gene is shown in Figure 4A derived from a three-way ANOVA comparison (STD, HFD and HFD+KAA). The genes are organized into rough groupings and are visualized as either orange (p<0.05) or blue (p>0.05) depending on their significance level (Table S6). Overall, the DNL pathway stands out as having a majority of genes significantly altered in their expression due to changing dietary composition. As for individual genes, the high-KAA diet repressed SREBP-1c but induced SHP expression (Figure 4B). Expression of other nuclear receptors including PPARα and LXRα did not change. In addition, down-regulation of SHP as well as up-regulation of SREBP-1c, FAS and SCD1 genes within the *de novo* lipogenesis pathway was clearly dependent on E/N ratio (Figure 4C).

### Effects of high-KAA diet on hyperinsulinemia and lipotoxic metabolite synthesis

**Figure 5:**
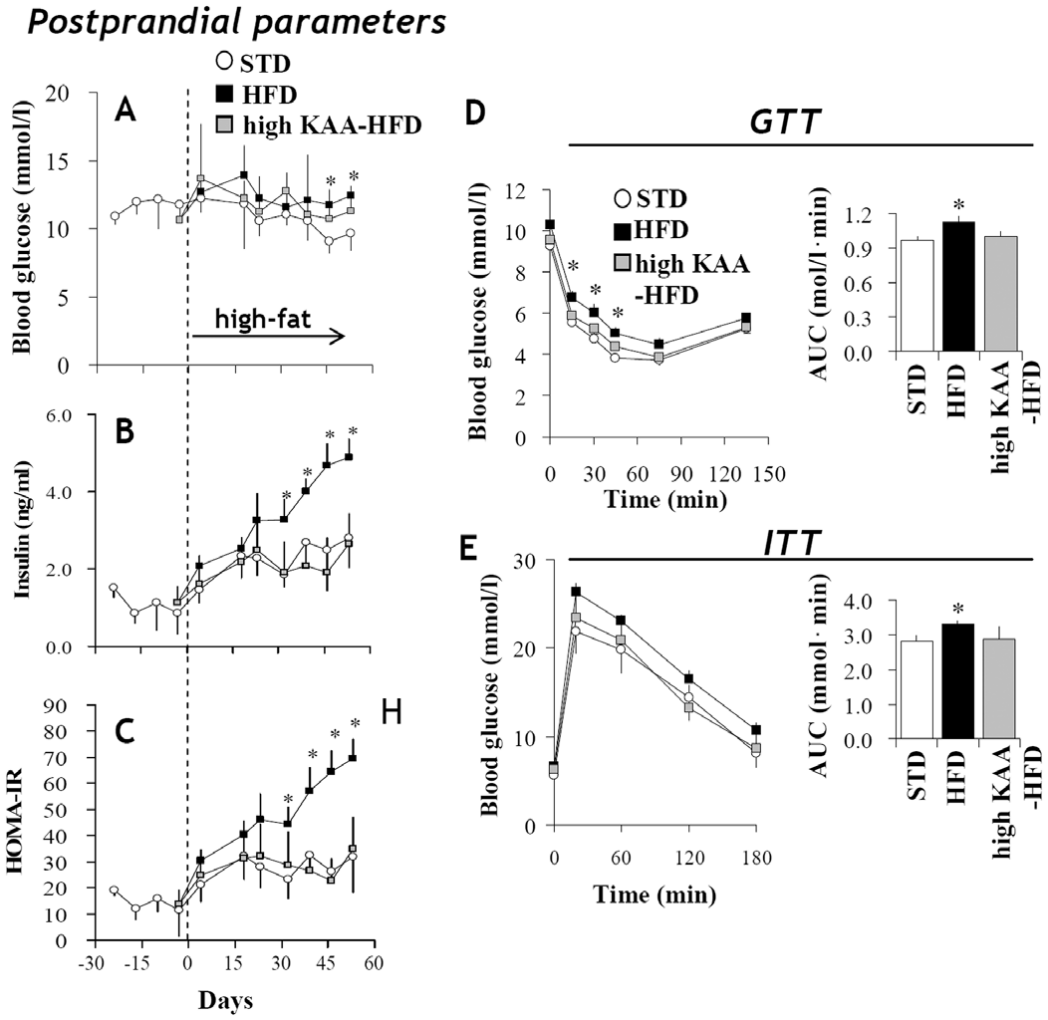
Attenuation of high-fat induced insulin resistance by dietary KAA. Plasma glucose (A), insulin (B) and calculated indices of HOMA-IR (C) in C57B6 mice 4 weeks before and 8 weeks after feeding with STD, HFD or high-E/N HFD (E/N = 1.8), under 3-hour fasting condition. Using C57B6 mice (n = 6 for each group) fed for 8 weeks with STD, HFD or the high-KAA (high E/N) diet (E/N = 1.8), glucose tolerance tests (GTT) (D), or insulin tolerance tests (ITT) (E) were performed by intraperitoneal glucose or insulin administration (at time = 0) after overnight fasting. *: p<0.05 for all high-fat groups as compared to STD group.

Long-term high-fat feeding is reported to induce insulin resistance in C57B6 mice [17]. Postprandial plasma glucose and insulin were measured throughout the 8-week experimental period. Though HFD feeding caused only slight increases in blood glucose in comparison to the STD group (Figure 5A), significant increases in both insulin levels and calculated HOMA-IR values were evident from 2 to 8 weeks (Figures 5B, C). However, the high-KAA diet fully normalized plasma insulin levels and HOMA-IR (Figures 5B, C). The high-KAA diet was shown to improve both glucose (GTT) and insulin tolerance test (ITT) response after 8 weeks of feeding, which were slightly impaired by HFD feeding (Figures 5D, E). Overall, the progression of insulin resistance caused by HFD feeding appears to be associated with the enhancement of hepatic DNL observed from 2 to 8 weeks (Figure 3C).

**Figure 6:**
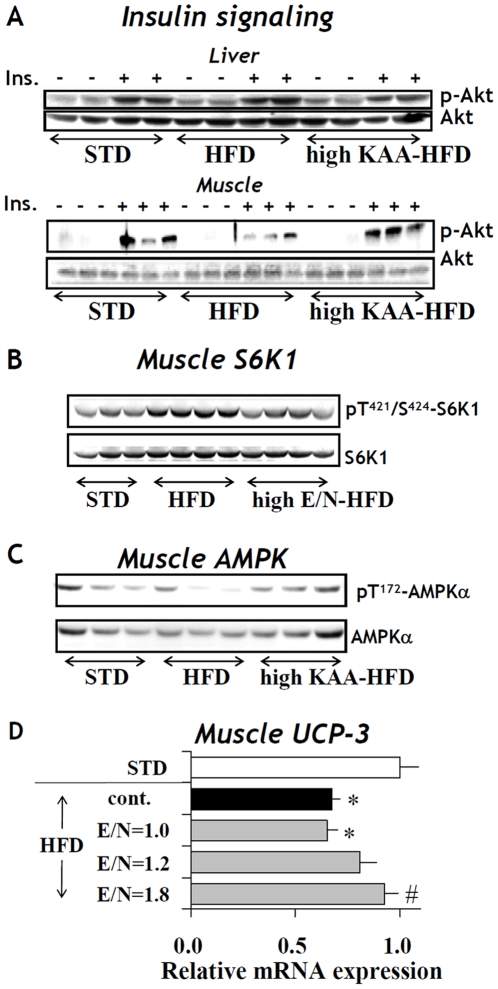
Dietary effects on phosphorylation and expression of metabolic regulatory proteins in muscle and liver. Samples were obtained from C57B6 mice (n = 3 for each group) fed for 12 weeks with STD, HFD or the high-KAA HFD (E/N = 1.8) except for (D), where mice were fed with the control HFD (cont.) or with HFD under varying E/N ratios. Total and phosphorylated Akt (p-Akt; Ser^473^) in the liver and muscle (A), S6K1 or S6K1-Thr^421^/Ser^424^ in the soleus muscle (B), and total and phosphorylated muscle AMPKα (C) were analyzed by Western blot. (D) Gene expression of UCP-3 in the soleus muscle was quantified by RT-PCR. Values are expressed as mean+/−SEM (n = 9). Symbols in (D) signify significant difference (p<0.05) for the comparison with STD (*) or control HFD (#).

Changes in insulin signaling in both liver and muscle were assessed by measuring insulin-induced Akt phosphorylation at 8 weeks. HFD feeding reduced Akt phosphorylation in muscle but not in liver, and the high-KAA diet normalized this effect in muscle (Figure 6A). Similarly, changes in muscular mTOR pathway signaling were probed by measuring insulin-induced phosphorylation of S6 kinase 1 (S6K1). HFD feeding augmented S6K1 and the high-KAA diet normalized this increase (Figure 6B). Further, muscular AMP phosphorylation and UCP-3 expression were reduced by HFD feeding, but dietary KAA fortification partially reversed this effect (Figures 6C, D).

**Figure 7:**
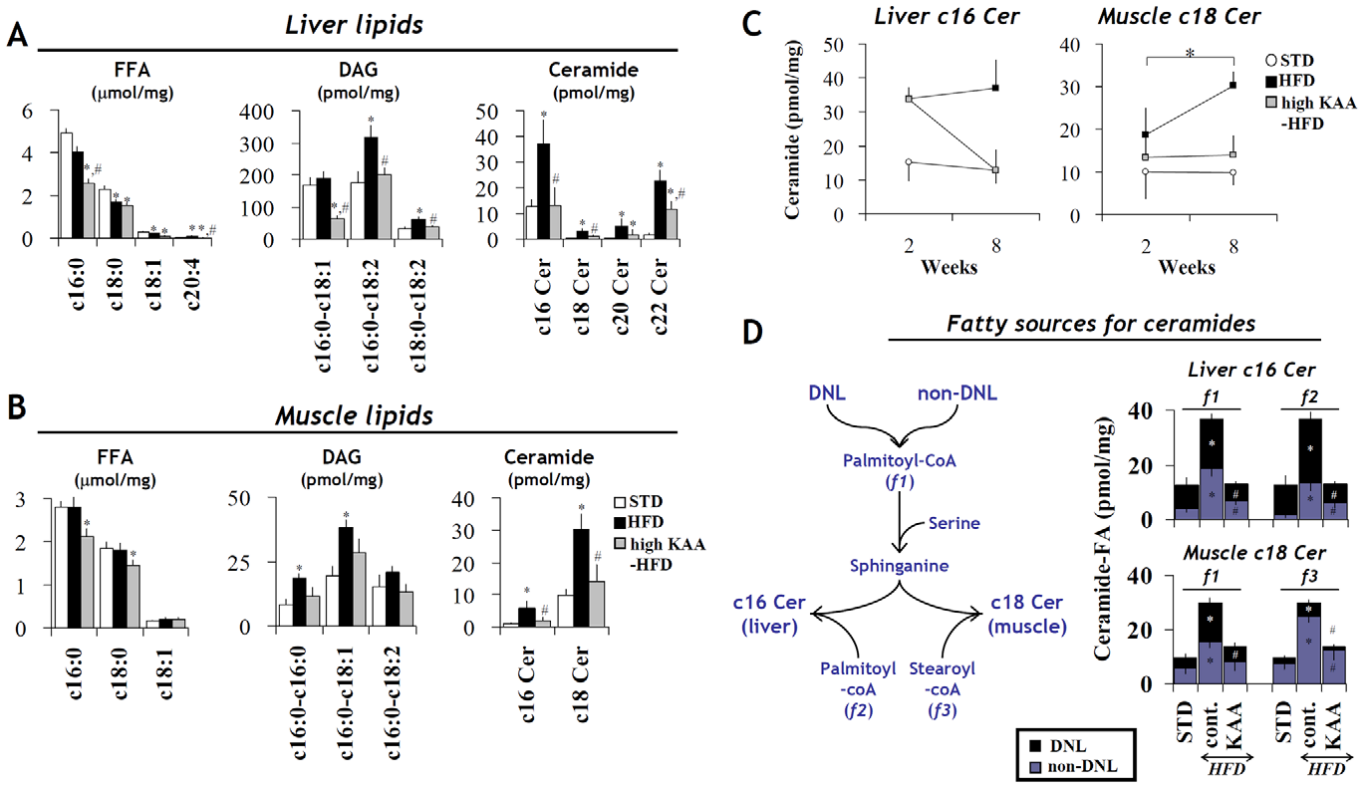
Effect of high-KAA diet on the production of hepatic and muscular lipotoxic metabolites. Samples were obtained from C57B6 mice (n = 6 for each group) fed for 8 weeks with STD, HFD or the high KAA HFD (E/N = 1.8), except for (C) where mice were sampled at 2 weeks in addition to 8 weeks. Lipid species of free fatty acids (FFA), diacylglycerols (DAG) and ceramides (Cer) in the liver (A) and gastrocnemius muscle (B) were quantified using GC-MS, where c16 Cer, c18 Cer, c20 Cer and c22 Cer correspond to palmitoyl-ceramide, stearoyl-ceramide, arachidyl-ceramide and docosanoyl-ceramide, respectively. Changes in liver c16 Cer and muscle c18 Cer concentrations were tracked between 2- and 8-week feeding periods (C). Metabolic pathway fluxes of hepatic and muscular ceramides were assessed by quantifying the contributions from palmitoyl-CoA (f1, f2) and stearoyl-CoA (f3) (see D left). The contribution of DNL-derived FA to ceramide synthesis was evaluated based on deuterium labeling of sphingosine- and acyl-groups (D), whereas non-DNL derived FA is illustrated with a blue column on the bottom of each bar. All values are expressed as mean+/−SEM (n = 6). *: p<0.05 for all high-fat groups as compared to STD group; #: p<0.05 for the high-KAA group as compared to HFD control.

Because lipotoxic metabolites such as ceramides and diacylglycerols (DAGs) are believed to be responsible for HFD-induced insulin resistance [4], GC-MS metabolite profiling was performed on liver and muscle biopsies. When compared with STD feeding, HFD feeding for 8 weeks increased DAG and ceramide concentrations in both tissues, while no obvious elevations were observed in FFA species (Figures 7A, B). The high-KAA diet prevented HFD-induced increases in ceramide and DAG levels and further lowered FFA concentrations relative to the control diet (Figures 7A, B). Stearoyl-ceramide (c18 Cer) and palmitoyl-ceramide (c16 Cer) were the major ceramide species in muscle and liver, respectively. It is noteworthy that HFD feeding increased muscular c18 Cer time-dependently, but not hepatic c16 Cer (Figure 7C). This sharp increase in muscular c18 Cer is associated with postprandial hyperinsulinemia shown in Figure 5A.

Stable isotope labeling experiments were applied to determine the relative contribution of DNL to each ceramide fatty-acyl group. As shown in Figure 7D, the extent of isotopic labeling in the sphingosine and fatty-acyl groups of ceramide molecules was analyzed, where *f1* denotes fatty acids incorporated into the sphingosine group, while *f2* and *f3* denote fatty acids incorporated into the fatty-acyl groups of hepatic c16 ceramide and muscular c18 ceramide, respectively. The results clearly show that both the sphingosine and fatty-acyl groups of liver c16 ceramide were mostly derived from DNL, whereas those of muscle c18 ceramide were derived from non-DNL sources, presumably dietary lipids. These data indicate that ceramide synthesis in the muscle relies primarily on dietary lipids, and therefore muscular ceramide content is expected to be more susceptible to the total content and feeding duration of dietary lipids. Thus, we hypothesize that if muscle is protected from accumulation of lipotoxic lipids, peripheral insulin resistance and postprandial hyperinsulinemia will improve.

### High-KAA diet prevents diet-induced hepatic steatosis in a hyperinsulinemic mouse model

**Figure 8:**
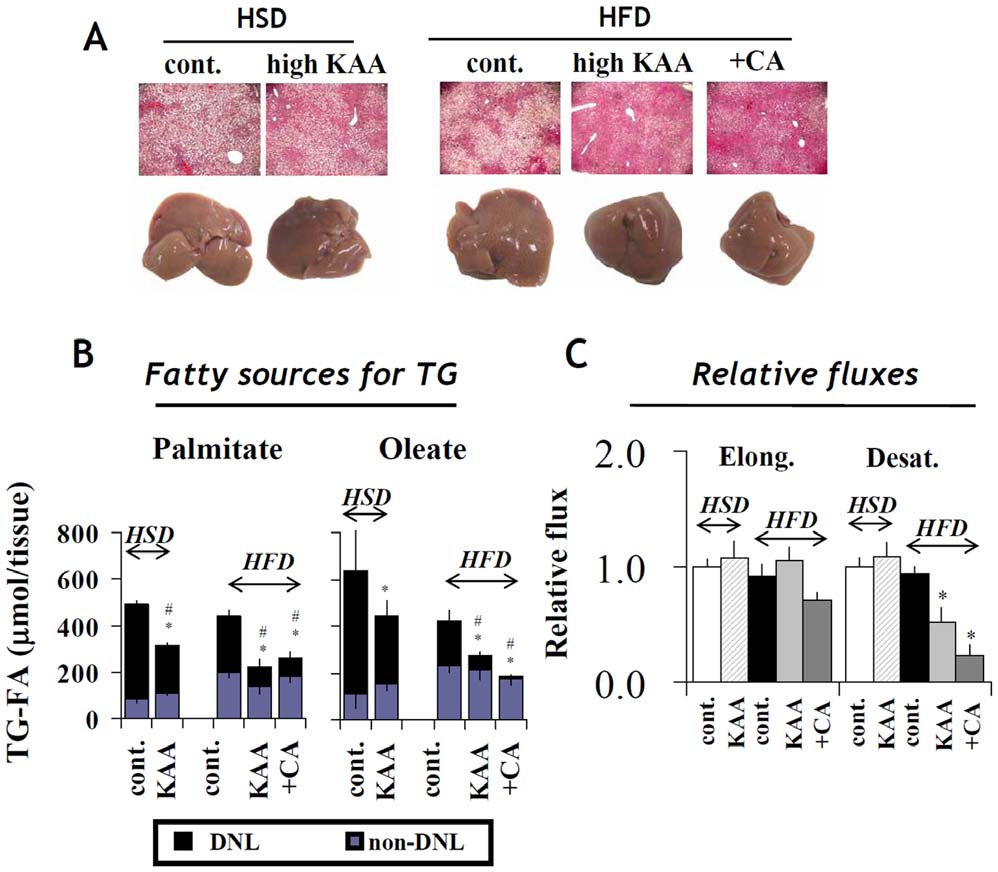
High dietary high KAA improves hepatic steatosis in hyperinsulinemic mouse model. Samples were obtained from *ob/ob* mice (n = 6 for each group) fed for 2 weeks with either a high-sucrose diet (HSD), or HFD in the presence (high E/N) or absence (cont.) of KAA fortification (E/N = 1.8) or cholate (+CA), which was used as an anti-hepatic-steatosis control agent. (A) Liver histology of hematoxylin-eosin staining (top) and oil red O staining (middle), and macroscopic liver appearances (bottom). (B,C) Metabolic fluxes of DNL pathways were analyzed by identifying fatty sources for hepatic triglycerides and estimating the *in vivo* relative contributions of fatty acid synthase (FAS), elongase and desaturase using deuterated water labeling and mass isotopomer distribution analysis (see Materials and Methods and Figure 3D). The contributions of elongase and desaturase (C) were assessed by DNL_c18:0_/DNL_c16:0_ and DNL_c18:1_/DNL_c18:0_, respectively. All values are expressed as mean+/−SEM (n = 6). In panel B, p<0.05 for DNL-derived FA and total FA versus the control group are indicated as * and #, respectively. In panel C, p<0.05 versus the control group is indicated as *.

To further understand the interactions between dietary amino acid composition and NAFLD development, we investigated the effect of a high-KAA diet on hepatic steatosis and insulin resistance in indigenously hyperinsulinemic *ob/ob* mice. The *ob/ob* mice were fed a HFD or high-sucrose diet (HSD) for 2 weeks. Cholate (CA) was administered as a positive control to suppress hepatic steatosis, since CA is known to inhibit hepatic DNL through FXR pathways [18], [19]. Both the high-KAA diet and CA administration separately improved hepatic steatosis (Figure 8A) and reduced liver weight and triglyceride content (Table 3) in response to either HFD or HSD. Although CA significantly increased hepatic cholesterol, the high-KAA diet had the opposite effect (Table 3). The high-KAA diet also reduced plasma GOT and GPT levels under either dietary condition, while CA led to marked increases in these parameters. Furthermore, the high-KAA diet did not induce changes in postprandial plasma insulin and glucose but partly reduced cholesterol and triglyceride levels under the HFD condition.

**Table 3:** Summary of metabolic parameters in *ob/ob* mice fed HSD or HFD with or without high E/N manipulation by KAA.

	HSD		HFD		HFD+CA
E/N ratio	0.8	1.8	0.8	1.8	0.8
	(n = 6)	(n = 6)	(n = 6)	(n = 6)	(n = 6)
Hepatic lipids					
Liver (g)	2.7±0.1	2.4±0.1^#^	2.7±0.1	2.2±0.0^#^	1.9±0.1^#^
Triglyceride (mg/g)	588±57	339±31^#^	529±56	285±28^#^	244±41^#^
Cholesterol (mg/g)	13.8±1.9	9.5±1.5^#^	12.1±2.5	5.5±0.4^#^	17.5±2.0^#^
Plasma parameters					
Glucose (mmol/l)	11.0±0.3	9.7±0.6	13.5±0.9	11.2±0.2	10.3±0.8^#^
Triglyceride (mg/dl)	90±5	87±5	132±7.5	104±2^#^	101±6^#^
Cholesterol (mg/dl)	227±5	223±10	240±17	211±6	179±10^#^
Insulin (ng/ml)	13.4±1.6	12.6±1.4	12.9±1.6	12.0±0.78	6.3±0.7^#^
GOT (unit)	230±30	155±9	141±26	75±4^#^	502±146^#^
GPT (unit)	270±29	196±10^#^	172±34	100±6	560±139^#^

Data represent mean +/− SE. *Ob/ob* mice were housed with indicated diets for 2 weeks. *, p<0.05 for all treatment groups with corresponding HFD or HSD control (E/N = 0.8).

Metabolic flux analysis clearly revealed that the high-KAA diet reduced hepatic DNL under HFD or HSD feeding, thus explaining the reduction in hepatic triglyceride-fatty acid content (Figure 8B). Under HFD but not HSD feeding, desaturase flux was decreased by either the high-KAA diet or CA administration (Figure 8C), similar to the observation in C57B6 mice shown in Figure 3D, but in this case elongase flux was not affected.

## Discussion

### Manipulation of dietary E/N ratio by ketogenic EAA

Bidirectional modifications of dietary protein content (restriction or excess) have been studied in terms of both their pathogenic and preventive effects on physiological alterations, such as insulin resistance and metabolic syndrome [20], [21]. Although classic studies showed an improvement in high-fat-induced metabolic changes by EAA supplementation in rodents [22], these findings have not been translated into practical measures for counteracting the growing tide of health problems associated with obesity and metabolic syndrome.

In most dietary proteins, EAA to NEAA ratios (E/N) stay within an almost constant range from 0.5 to 0.8 [23]. NEAAs primarily consist of glucogenic amino acids such as glutamine and aspartate, while EAAs consist of ketogenic amino acids such as BCAAs and lysine. Therefore, several points should be considered with regard to dietary KAA supplementation. First, an increase in protein content via a natural diet achieves higher KAA intake but also results in simultaneously higher glucogenic amino acid intake, which is often associated with high fat intake. Second, from a practical viewpoint, the amount of dietary protein should rather be managed downward in a diabetic or pre-diabetic condition, because renal failure is one of the common diabetic complications [24]. Third, in animal experiments amino acids or KAA are often loaded through drinking water, which does not result in consistent changes in the balance between amino acids and lipids. Fourth, anorexic effects of KAA loading must be considered, particularly to rodents. The accompanied decrease in calorie intake would distort metabolism as a whole, making the interpretation of experimental results difficult. Leucine, methionine and histidine have been characterized as having a robust influence on food intake in rodents [25]. Among these, leucine was reported to stimulate mTOR signaling directly in the hypothalamus, leading to decreased food intake [26], [27] . Lastly, amino acid imbalance caused by supplementing either a single amino acid or a few specific amino acids will negatively impact the so-called “metabolic value” of the diet in question. Taken together, these factors have largely confounded previous attempts to assess the direct or upstream metabolic effects of KAAs.

In our preliminary experiments, we did not observe a reduction in food intake associated with feeding a mixture of selected KAAs to rats under a high-fat diet condition, suggesting that utilization of multiple KAAs together could alleviate the potential for anorectic effects. In contrast, addition of methionine or histidine to the KAA mixture effectively worsened its anorectic effect. Thus, we formulated our KAA diet by replacing part of the natural casein-derived protein with a KAA mixture that excluded the “anorectic” methionine and histidine. This mixture was composed of free BCAAs plus lysine and threonine, which are quantitatively the major KAAs in most animal proteins. This enabled us to achieve loading of multiple KAAs and a high E/N ratio, while avoiding changes to dietary fat and carbohydrate intake as well as total amino acid intake. Although further optimization of our formulation will be required to elucidate the contribution of each individual amino acid, the KAA diet developed in the present study exhibits a marked capacity to modulate high-fat-induced metabolic alterations and to prevent subsequent pathogenesis.

### Effect of high KAA diet on hepatic steatosis

Hepatic steatosis can be induced by various nutrient conditions such as high-fat overfeeding or KAA deprivation [7], [28], suggesting metabolic interactions between dietary fat and KAA intake. The present study provided evidence that a high-KAA diet can prevent high-fat-induced hepatic steatosis. Hepatic lipids are generally considered to be supplied from extra-hepatic lipids under high-fat feeding. In contrast, the major source of hepatic lipids under high-carbohydrate feeding is hepatic *de novo* lipogenesis (DNL) [29]. Induction of fatty liver in C57B6 mice usually requires dietary manipulation of lipids or carbohydrate. On the other hand, *ob/ob* mice spontaneously develop fatty liver, though similar dietary manipulations can facilitate fatty liver formation and induce more severe pathogenesis. In the present study, we applied a fortified E/N diet based upon KAA supplementation to significantly lower hepatic lipids in both wild-type and *ob/ob* mice. Because *ob/ob* mice were fed either a high-fat or high-sucrose diet, KAA supplementation apparently suppressed not only DNL but also lipid translocation from extra-hepatic sources. To confirm this, we applied metabolic flux analysis to quantify DNL in both liver and fat tissues of *ob/ob* mice. Our analysis confirmed that hepatic DNL was lower in the HFD condition compared to the HSD condition. Unexpectedly, hepatic DNL increased in a time-dependent manner (2- vs 8-week feeding) in C57B6 mice fed with HFD and became the major contributor to hepatic TG, although non-DNL sources of fatty acids also increased slightly over this period. The high-KAA diet consistently suppressed hepatic DNL in both mouse strains (C57B6 and *ob/ob*) and under both dietary conditions (HFD and HSD) tested in this study, demonstrating that the reduced hepatic TG content observed was mainly due to suppression of DNL. Further, the high-KAA diet repressed liver expression of lipogenic genes such as FAS and SCD1 in an E/N-dependent manner. Interestingly, this repression in *ob/ob* mice appeared to be mediated by the SREBP-1c pathway in the HFD condition but through the ChREBP pathway in the HSD condition (Figure S2). Underscoring this hypothesis, liver pyruvate kinase (L-PK), which is regulated by the ChREBP but not SREBP-1c pathway, was shown to be repressed with the high-KAA diet under HSD feeding. Furthermore, up-regulation of SHP by the high-KAA diet in both strains of mice under HFD feeding can be reasonably understood because SHP is regulated by FXR and can act as a suppressor of SREBP-1c [18]. The precise mechanism by which KAAs modify the expression of these nuclear receptors is yet to be elucidated. Further metabolite profiling will be needed to examine specific KAA metabolites, such as keto-acyl-CoAs, which may affect the expression of lipogenesis-related nuclear receptors.

In C57B6 mice, the high-KAA diet increased plasma β-hydroxybutyrate (β-OH-butyrate). However, there was no corresponding increase in liver expression of genes involved in fatty acid oxidation. As mentioned, all KAAs used in the present study generate keto-acyl-CoA in the liver. It is well-known that β-OH-butyrate can be formed from leucine, isoleucine and lysine in addition to fatty acids. In the present study, however, the precise carbon source of plasma β-OH-butyrate is not clear, and it is possible that increased hepatic lipid oxidation may also contribute to the reduction of hepatic TG observed on the high-KAA diet.

### Is insulin resistance a prerequisite to liver steatosis

Lipid profiling of liver and muscle in the present study revealed that both DAG and ceramide concentrations were lowered by the high-KAA diet. DAG is considered a primary inducer of insulin resistance in response to excess unsaturated fat feeding [30], [31], while ceramide is synthesized directly from saturated fatty acids and antagonizes insulin action in muscle [4]. Lard was used as the primary dietary fat source in the present study, and thus both palmitate and oleate, the major constituents of lard, would contribute to the induction of insulin resistance. DNL-derived fatty acids were found to contribute more to ceramide synthesis in liver than in muscle, but a significant time-dependent increase in muscle ceramide was observed between 2 and 8 weeks of HFD feeding. Liver ceramide levels are expected to be in dynamic equilibrium with other lipid species, as enhanced triglyceride synthesis may compete directly with ceramide synthesis and increased secretion of lipoproteins is expected to decrease ceramide levels. Stable isotopic flux analysis revealed that muscle ceramides relied heavily on dietary lipids as their FFA source. In addition, our data show that DNL contributed relatively less stearoyl- than sphingosine-moiety to muscle c18 ceramide, suggesting that low SCD1 activity in muscle could be responsible for the increased dependency of muscle ceramide accumulation on dietary fat. Therefore, our present finding of reduced muscular c18 ceramide accumulation in response to a high-E/N diet could be attributable to the reduced conversion of both DNL-derived palmitate into the sphigosine-group and dietary lipids into the stearoyl-group of c18 ceramide. In a previous study, Monetti et al.[32] report that various lipids, including TG, DAG and ceramides, accumulated in mouse liver as a result of overexpression of acyl-CoA:diacylglycerol acyltransferase (DGAT) but without associated insulin resistance, and they concluded that hepatic steatosis could ensue independently of insulin resistance. Further, another report [33] illustrates that muscular insulin resistance is directly related to reduced muscular glucose utilization and hepatic steatosis. Furthermore, it has been reported that elevated muscular ceramides, but not hepatic ceramides, lead to insulin resistance [32]. Thus, muscle appears to be more susceptible to lipotoxicity than liver, and readily develops an insulin resistant state. Based on those reports, the prevention of hepatic steatosis by high KAA intake could be at least partly attributed to protection against peripheral insulin resistance and enhanced glucose utilization by the liver. Indeed, our results indicate that, even when insulin signaling in the liver remained intact, muscle insulin signaling was more easily impaired by HFD feeding.

The high-KAA diet increased muscular UCP-3 expression under HFD conditions, which could lead to enhanced energy expenditure associated with increased fatty fuel oxidation. Choi et al.[34] claim that overexpression of muscle UCP-3 in mice protected them from HFD-induced insulin resistance, and Zhang et al.[14] report that leucine loading in mice increased muscular and fatty UCP-3 expression. The precise mechanism linking amino acid supplementation with UCP-3 expression, however, remains elusive. High-fat feeding was reported to reduce muscle AMP-activated protein kinase (AMPK), a possible UCP-3 regulator [35], and muscular AMPK reduction is expected to decrease glucose disposal. A few studies along these lines suggested a causal relationship between lipotoxic metabolites and decreased AMPK phosphorylation [36], [37]. In agreement with this hypothesis, our data demonstrate that high KAA intake reversed the inhibition of AMPK phosphorylation in the presence of a high-fat diet. Thus, the reduction of lipotoxic lipids could be one of the major pathways by which the high-KAA diet maintains muscle AMPK and extrahepatic substrate utilization.

The high-KAA diet was fed to leptin-deficient *ob/ob* mice that had already developed noticeable insulin resistance and hepatic steatosis before KAA treatment. As a result, 2 weeks of feeding significantly reduced hepatic lipids, though hyperinsulinemia evidently still remained. Thus, the acute reduction of hepatic lipids by the high-E/N diet seems to occur through independent mechanisms of preventing peripheral insulin resistance in C57B6. Although it was not clear why the high-E/N diet did not affect postprandial insulin levels in *ob/ob* mice, muscle lipotoxic lipids were considerably higher before KAA treatments than those in high-fat treated C57B6 mice. Therefore, this may explain why short-term feeding of the high-E/N diet was not sufficient to reduce lipotoxic lipids to the point where peripheral insulin resistance was restored. Though further study is to be done, altered expression of nuclear receptors such as SREBP-1c and SHP by the high-E/N diet should be at least one of the factors contributing to an acute reduction of hepatic lipids.

### Protein overfeeding and KAA fortification

Protein and lipid overfeeding is reported to induce an insulin resistant state depending on the ribosomal protein S6 kinase 1 (S6K1) and its effector, mammalian target of rapamycin (mTOR) pathway [11]. We found that a high-protein diet induces global elevation of KAA levels [38], [39] . Elevation of circulating amino acids such as alanine and BCAAs has been recognized as a marker of protein overfeeding and is often associated with an insulin resistant state, for instance, in obese individuals [40], [41]. Activation of S6K1 and mTOR expressions were confirmed in human muscle by simulation of protein overfeeding with a constant infusion of 20 proteinogenic amino acids [12], [41]. Because our high-KAA diet neither generated elevated plasma amino acids (Table S4) nor activated the expression of muscular S6K, the metabolic impact of KAA fortification appears fundamentally different from protein overfeeding.

### Conclusion

In summary, we show that dietary amino acid manipulation, in which protein is partially replaced by free ketogenic essential amino acids, can modulate metabolic alterations and prevent hepatic steatosis in mice models of diet-induced obesity. We conclude that increasing dietary ketogenic amino acids may offer a new preventive and therapeutic approach to address non-alcoholic fatty liver disease.

## Materials and Methods

### Animals

All studies were reviewed and approved by the Animal Care Committee of Ajinomoto Co., Inc. and Massachusetts Institute of Technology. Ten-week-old male C57B6 mice were obtained from Charles River Laboratory, Japan Inc. and Taconic Farms Inc. Ten-week-old male *ob/ob* mice were obtained from Jackson Laboratory. All mice were housed in colony cages, maintained on a 12∶12-hour light and dark rhythm with free access to water. Blood was collected in tubes on ice containing ethylenediaminetetraacetatic acid (EDTA; NONCLOT-D, Daiichi Pure Chemicals, Tokyo, Japan). Liver, epididimal fat and gastrocnemius muscle were collected for lipid and gene expression analyses. Soleus was collected for western-blot analysis. All collected tissues were immediately clamped into liquid nitrogen and stored at −80°C.

### Diets

For C57B6 mice experiments, standard diet (STD), high-fat diet (HFD) and KAA-fortified HFD (HFD+KAA) were prepared based on the AIN-93G composition (Table S1, S2). The casein-mimic free amino acid mixture (CAAM) or KAA mixture was used to replace the part of protein (Table S1, S2). Using partial protein replacement by free KAA, E/N ratio in diet was graded from 0.8 (control) to 1.8, where to avoid changes in amounts of dietary total amino acids, fat and carbohydrates, total amino acids including protein-amino acids were equalized among the groups by adding above mentioned CAAM up to that of 23%, if necessary. For *ob/ob* mice experiment, high-sucrose diet (HSD) and HFD were prepared in the presence or absence of KAA fortification (E/N = 1.8). Cholate (CA) was used as a positive control of anti-hepatic steatosis agent, because CA was known to repress hepatic DNL through FXR pathways [18], [19]. We also purchased STD, HFD and HFD+KAA from Research Diet Inc.(New Brunswick, NJ) for tissue lipid analyses (Table S3).

### Energy expenditure

Energy expenditure (EE) of individual mice was measured using indirect calorimetry. An animal was housed in a metabolic cage for 24 h allowing collection of urine and feces separately [16]. Food was available between 1900 and 0800 h. Oxygen consumption and CO_2_ production were determined every 5 min in an open chamber with a mass spectrometry based O_2_ and CO_2_ analyzer ARCO-2000 (ARCO system, Chiba, Japan). Oxygen consumption was normalized by lean body mass.

### Blood biochemistry

Blood glucose, cholesterol and triglyceride were measured using DRI-CHEM5500 (FUJI films, Tokyo, Japan). Plasma free fatty acids were determined using an enzymatic method by an automated kit according to the manufacturer's specifications (Wako Pure Chemical Industries Ltd, Osaka, Japan). Ketone bodies were measured by a ketone test kit (Sanwa Kagaku Kenkyusho Co., Ltd, Nagoya, Japan). Serum leptin and insulin were determined using commercial mouse ELISA kits (Seikagaku-kogyo Co., Tokyo, Japan). Plasma sphingomyelin was analyzed using a commercial kit (Cayman Chemical, Ann Arbor, Michigan, USA).

Mouse lipoproteins were prepared by FPLC analysis of plasma using a Superose 6 column (GE healthcare) on a FPLC system model 600 from Waters as described previously [42]. A 100-µl aliquot of pooled plasma from each group was injected onto the column and separated with a buffer containing 0.15 M NaCl, 0.01 M Na_2_HPO_4_, and 0.1 mM EDTA at pH 7.5 using a flow rate of 0.5 ml/min. Fifty fractions of 0.5 ml each were collected, with the lipoproteins contained in tubes 15–33. Fractions 15–19 = very-low-density lipoprotein and chylomicrons; fractions 20–26 = intermediate -density lipoproteins, low-density lipoproteins, and large high-density lipoproteins; fractions 27–33 = high-density lipoprotein.

### Glucose and insulin tolerance tests

For glucose tolerance tests, blood glucose was measured at 0, 30, 60, 120 and 150 min after a bolus intraperitoneal glucose administration (1 mg/g body wt) to overnight-fasted mice. For insulin tolerance tests, regular human insulin was administered intraperitoneally (0.75 mU/g) to mice after 4-h food deprivation, and blood glucose was measured at 0, 30, 60, 120 and 150 min after insulin injection. The homeostasis model assessment of insulin resistance (HOMA-IR) index was calculated based on the conventional formula: HOMA-IR = basal glucose (mmol/l) × basal insulin (mU/l)/22.5.

### Western blot

Liver and muscle tissue (50–100 mg) was homogenized in a detergent-based lysis buffer (M-PER; Pierce Biotechnology) supplemented with protease and phosphatase inhibitors. The extracts were incubated on ice for 20 minutes and then centrifuged at 15,000 g to remove tissue debris. The supernatants were saved and frozen at −80°C until analysis. Then, 50 µg of protein were heat denatured in SDS-PAGE sample buffer and resolved on denaturing (SDS) 10% polyacrylamide gels (SDS-PAGE). Fractionated proteins were electrophoretically transferred to nitrocellulose membranes by standard procedures. Immunoblotting was carried out using the following antibodies: anti-p-Akt (Ser473) (1∶1000 dilution, Cell Signaling Technology), anti-p-S6K1 (Thr421/Ser424) (1∶1000 dilution) and anti-p-AMPKα (Thr172) (1∶1000 dilution, Cell Signaling Technology) as indicated in Figure 6. Membranes were immunoblotted with the following primary antibodies. Immune complexes were detected by luminescent image analyzer LAS-3000 (Fujifilm, Tokyo, Japan).

### Histology

Formalin-fixed tissues were embedded in paraffin using standard procedures. Sections at 4µm thick were stained with Hematoxylin-eosin for general observation, and frozen sections at 5µm thick were stained with Oil Red O and counterstained with Hematoxylin for visualizing lipids.

### Real-time PCR

Total RNA was extracted from the homogenized liver using an RNAeasy kit (Qiagen, Germantown, MD) following the manufacturer's instructions. mRNA was then extracted from total RNA preparations using an Oligotex kit (Qiagen) following the manufacturer's instructions. Quality and integrity of the RNA were checked by A_260_/_280_ ratio and on a formaldehyde/agarose gel, respectively. Equal amounts of RNA were reverse transcribed using Superscript II reverse transcriptase (Invitrogen, Carlsbad, CA) as per the manufacturer's instructions. Primers for RT-PCR were designed using the primer design software Primer3 (Table S5), and then the sequence homology for related proteins was checked. 18S ribosomal RNA primers were used as an endogenous control. RT-PCR was performed on an ABI Prism® 7700 Sequence Detection System (PE Applied Biosystems, Foster CA), and the data obtained were analyzed using the provided software. The reaction mixture consisted of 4 µl cDNA template, 10 µl of Sybr Green PCR master mix (Roche Biochemicals, IN), 2 µl of 0.25–1 µM forward primer, and 2 µl of 0.25–1 µM reverse primer in a 20 µl reaction volume. The PCR protocol consisted of one 10 min denaturation cycle at 95°C followed by 40 cycles of denaturation at 95°C for 15 sec and annealing/extension at 60°C for 1 min. Standard curves for each gene and endogenous 18S ribosomal RNA control were obtained. The efficiency of PCR amplification was 100%, and the *R* ^2^ value was between 0.995 and 0.999. All RT-PCR data were expressed as relative mRNA levels after normalizing to 18S ribosomal RNA.

### Plasma amino acids profiling

Plasma samples and tissues were treated with 2 volumes of 5% (w/w) trichloroacetic acid (TCA) and then centrifuged to remove protein as precipitate. The samples obtained were filtered through an Ultrafree-MC centrifugal filter (Millipore, Billerica, MA). All samples were kept at 4°C during all steps to minimize chemical reactions of thiol-metabolites and stored at −80°C. The amino acid concentrations were measured by an automatic amino acid analyzer (L-8800, Hitachi, Tokyo, Japan). Briefly, amino acids separated by cation-exchange chromatography were detected spectrophotometrically after post-column reaction with ninhydrin reagent.

### Tissue lipid analysis

For tissue metabolite extraction, we employed a biphasic extraction protocol, with non-polar metabolites partitioning into a chloroform phase and polar metabolites partitioning into a methanol/water phase as described previously[43]. Frozen 50∼100 mg of liver, muscle and fat tissues were homogenized (Polytron, Brinkmann Instruments) in 2 ml ice-cold methanol∶water (1∶1, v/v) containing 0.2 mg butylated hydroxytoluene as antioxidant. Afterwards, 15.8 nmol triheptadecanoin, 2.94 nmol 5α-cholestane and 3.5 nmol N-acetyl-sphingosine (C2 ceramide), 2.25 nmol heptadecanoyl-sphingosine (C17 ceramide) and 7.4 nmol 1, 3-dipendecanoin in 30 µl chloroform (non-polar internal standards) and also 9.9 nmol ribitol and 11.5 nmol norvaline in 30 µl methanol (polar internal standards) were added. After addition of 1 ml chloroform, samples were shaken for 30 min at room temperature and then 3 ml chloroform and 2 ml water were added. Vortexed samples were centrifuged at 4,000g for 30 min at room temperature. Two 2 ml extracts from the methanol/water phase or non-polar samples from the chloroform phase were separately collected to new tubes and then evaporated to dryness. All samples were stored at −80°C while awaiting analysis.

For determination of non-triglyceride lipids such as FFA, diacylglyerols, and ceramides, dried non-polar samples were dissolved in 1.55 ml of isooctane: methanol: ethyl acetate (20∶10∶1). To remove triglyceride, samples were applied to a silica gel packed Poly-Prep column (BIO-RAD, Hercules, CA, USA) as previously described [44], [45]. Eluted free lipid fractions were evaporated to dryness. Samples were dissolved in 150 µl BSTFA+1% TMCS: acetonitrile (4∶1) and then incubated overnight at room temperature. GC-MS analysis of lipid species was performed with the following parameter settings. The temperature of the injection port, MS source and quadrupole were set at 310°C, 230°C and 150°C, respectively. The GC temperature program was set as follows: 3 min at 130°C, 4 min ramp to 190°C, 3 min at 190°C, 12.3 min ramp to 264°C, 5 min at 264°C, 5.75 min ramp to 287°C, 8 min at 287°C, 4.6 min ramp to 310°C, 3 min at 310°C, 4.7 min ramp to 325°C, and 16.6 min at 325°C (total 70 min per run).

For fatty acids analysis in cellular lipids, dried non-polar samples were dissolved in 250 µl of 0.5 N KOH in methanol and then incubated at 70°C for 1h. After addition of 250 µl of 14% BF_3_ in methanol, samples were further incubated at 70°C for 2h. 250 µl saturated NaCl water was added to the resulting samples, and fatty acid methyl esters were extracted twice in 500 µl hexane. The temperature program for fatty acid methyl esters was set as follows: 5 min at 100°C, 5 min ramp to 175°C, 1 min at 175°C, 11 min ramp to 208°C, 3.6 min at 208°C, 1.4 min ramp to 215°C, 4 min at 215°C, 2 min ramp to 215°C, 7 min ramp to 255°C, 3 min ramp to 300°C, and 2 min at 300°C (total 45 min per run).

### GC-MS data analysis

GC-MS data were analyzed according to Styczynski et al. [46]. Briefly, mass spectra were processed by AMDIS software (http://hemdata.nist.gov/mass-spc/amdis/ National Institute of Standards and Technology). The resulting ELU files were further analyzed by the SpectConnect software (http://spectconnect.mit.edu/) developed in our lab to identify well conserved peaks among multiple GC-MS chromatograms. Metabolite identification of EI-MS peaks was performed using in-house standard libraries along with the NIST05 MS library.

### Determination of fatty source for triglyceride and ceramide synthesis

Deuterated water (D_2_O; Aldrich) was provided as deuterium source for incorporation into fatty acid (FA) during *de novo* lipogenesis and ceramide synthesis. An initial priming dose of D_2_O (4% body weight) by i.p. injection was followed by a maintenance dose of 6% (vol/vol) D_2_O in drinking water. After 7 to 9 days, tissue samples were collected. Deuterium enrichment in body water was determined by GC-MS as previously described [47] Total lipid was isolated from both liver, fat and muscle samples after saponification. The isolated FAs were methylated and analyzed by GC-MS as described above. Mass isotopomer distributions were determined using the method of Lee et al., which corrects for the contribution of derivatizing agent and ^13^C natural abundance [48]. The spectra of the palmitate (270–276 *m/z*), stearate (297–304 *m/z*) and oleate (263–276 *m/z*) peaks were analyzed for their isotopomer distribution and deuterium contents, which were used to calculate the fraction of newly synthesized FA. Estimations of chain elongation to stearate and of desaturation to oleate were performed as described previously[48], [49]. To estimate *de novo* ceramide synthesis, mass isotopomer distributions were determined in both sphingosine- and acyl-moieties (Figure S3). The spectra of palmitoyl-ceramide (311–317 *m/z* for sphingosine; 370–376 *m/*z for acyl-group) and docosanoyl-ceramide (311–317 *m/z* for sphigosine; 454–460 *m/z* for acyl-group) in liver and stearoyl-ceramide (311–317 *m/z* for sphigosine; 398–404 *m/z* for acyl-group) in muscle were analyzed.

### Statistics

Data are presented as means ± SEM unless otherwise indicated. Data were analyzed by one-way ANOVA using Tukey's post hoc test to determine statistical significance for all pairwise multiple comparison procedures and Dunnett's test for multiple comparisons against the control group.

## Supporting Information

Table S1 Diet composition. Casein mimic AA mixture containing the following percentages: 2.5% His, 4.5% Phe, 8.8% Lys-HCl, 1.1% Trp, 3.8% Thr, 2.4% Met, 4.5% Ile, 5.7% Val, 8.1% Leu, 9.4% Pro, 9.4% Asn.H2O, 4.9% Tyr, 2.6% Ala, 3.3% Arg, 5.1% Ser, 9.2% Glu, 9.2% Gln, 1.6% Gly, 0.5% Cystine, 3.2% Asp and 6.2% starch, respectively.
(0.09 MB TIF)

Table S2 Diet composition.Casein mimic AA mixture containing the following percentages: 2.5% His, 4.5% Phe, 8.8% Lys-HCl, 1.1% Trp, 3.8% Thr, 2.4% Met, 4.5% Ile, 5.7% Val, 8.1% Leu, 9.4% Pro, 9.4% Asn.H2O, 4.9% Tyr, 2.6% Ala, 3.3% Arg, 5.1% Ser, 9.2% Glu, 9.2% Gln, 1.6% Gly, 0.5% Cystine, 3.2% Asp and 6.2% starch, respectively.
(0.07 MB TIF)

Table S3 Diet composition.
(0.08 MB TIF)

Table S4 Plasma amino acids from c57B6 mice fed HFD for 8 weeks. Data represent mean+/−SEM (n = 6). *, p<0.05 for all high-fat groups compared with STD group; #, p<0.05 for high E/N groups with high fat control.
(0.12 MB TIF)

Table S5 Primer sequences used for RT-PCR.
(0.47 MB TIF)

Table S6 Hepatic gene expressions in C57B6 mice. a, Genes are categorized based on KEGG metabolic pathway database. Each letter means the following metabolic pathways: A, amino acid metabolism; L, Lipid metabolism; C, carbohydrate metabolism inculiding glycolysis, gluconeogensis, TCA cycle, glycogen metabolism and pentose phosphate pathway. Data represent mean+/−SEM (n = 9).
(0.17 MB TIF)

Figure S1 Influences of dietary protein level on metabolic parameters both in low and high fat diet. C57B6 mice were housed with diet containing indicated percent of casein for 8 weeks. Data represent mean+/−SEM (n = 6). *, p<0.05 versus 20% casein group.
(0.81 MB TIF)

Figure S2 Hepatic expression analysis of lipogenic genes in *ob/ob* mice. Data represent mean+/−SEM (n = 6). *, p<0.05 for the treatment groups as compared to control or as indicated.
(0.44 MB TIF)

Figure S3 Stable isotopic analysis of ceramide acyl-and sphigonsine groups. Molecular fragments and ions of acyl -and sphingosine groups in TMS-derivatized ceramides (A) and their mass spectra (B).
(0.59 MB TIF)
