# Supplementary figures and images for: Ketogenic Essential Amino Acids Modulate Lipid Synthetic Pathways and Prevent Hepatic Steatosis in Mice
Source: PLoS One. 2010 Aug 10;5(8):e12057. doi: 10.1371/journal.pone.0012057 (PMC2919399; doi:10.1371/journal.pone.0012057)

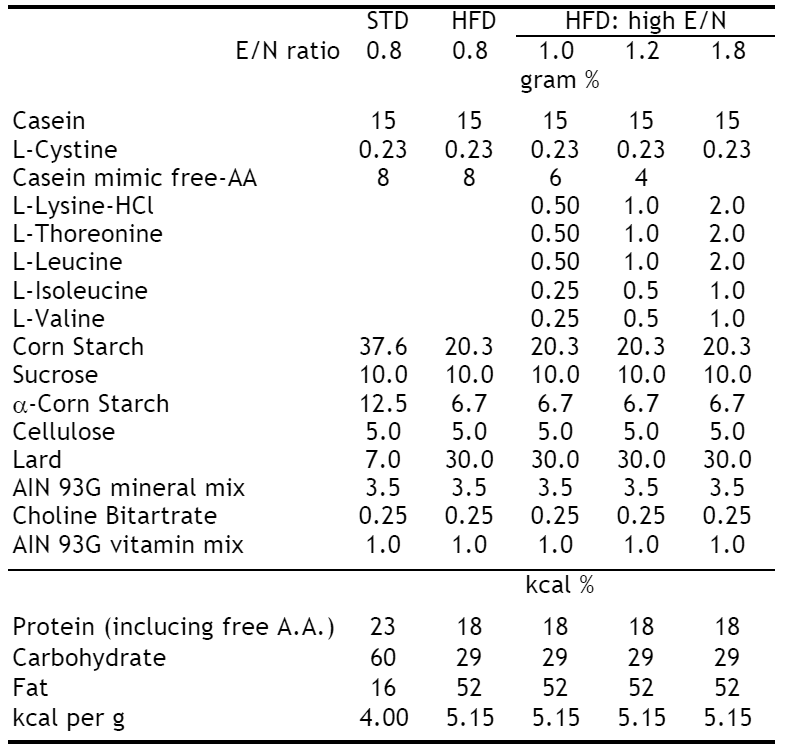

Supplement: Table S1 — Diet composition. Casein mimic AA mixture containing the following percentages: 2.5% His, 4.5% Phe, 8.8% Lys-HCl, 1.1% Trp, 3.8% Thr, 2.4% Met, 4.5% Ile, 5.7% Val, 8.1% Leu, 9.4% Pro, 9.4% Asn.H2O, 4.9% Tyr, 2.6% Ala, 3.3% Arg, 5.1% Ser, 9.2% Glu, 9.2% Gln, 1.6% Gly, 0.5% Cystine, 3.2% Asp and 6.2% starch, respectively. (0.09 MB TIF) [file pone.0012057.s001.tif]

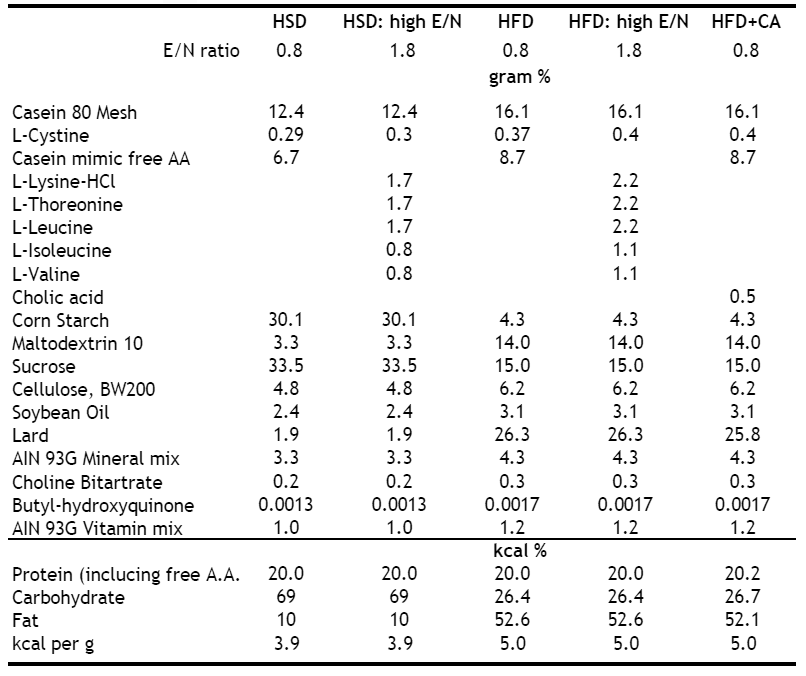

Supplement: Table S2 — Diet composition.Casein mimic AA mixture containing the following percentages: 2.5% His, 4.5% Phe, 8.8% Lys-HCl, 1.1% Trp, 3.8% Thr, 2.4% Met, 4.5% Ile, 5.7% Val, 8.1% Leu, 9.4% Pro, 9.4% Asn.H2O, 4.9% Tyr, 2.6% Ala, 3.3% Arg, 5.1% Ser, 9.2% Glu, 9.2% Gln, 1.6% Gly, 0.5% Cystine, 3.2% Asp and 6.2% starch, respectively. (0.07 MB TIF) [file pone.0012057.s002.tif]

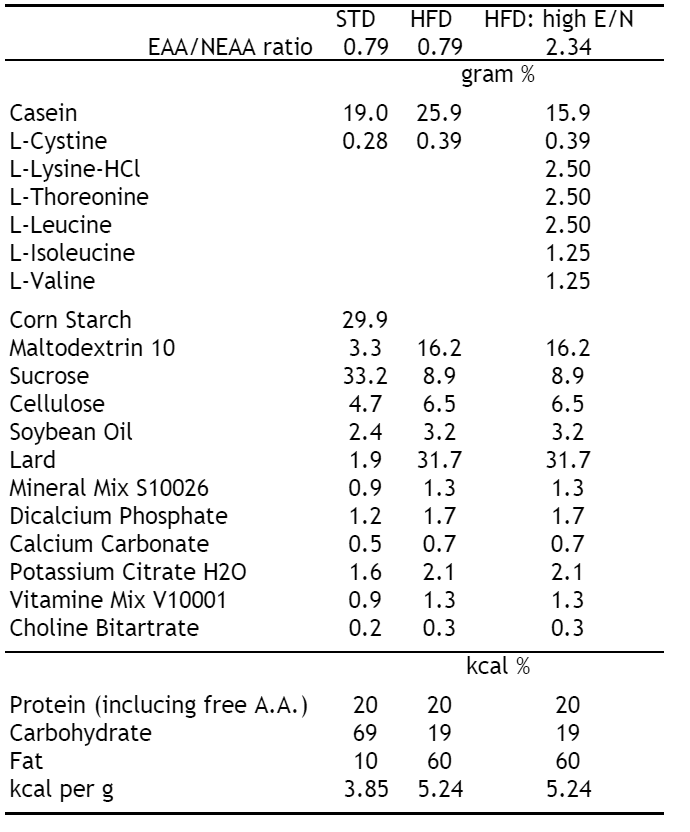

Supplement: Table S3 — Diet composition. (0.08 MB TIF) [file pone.0012057.s003.tif]

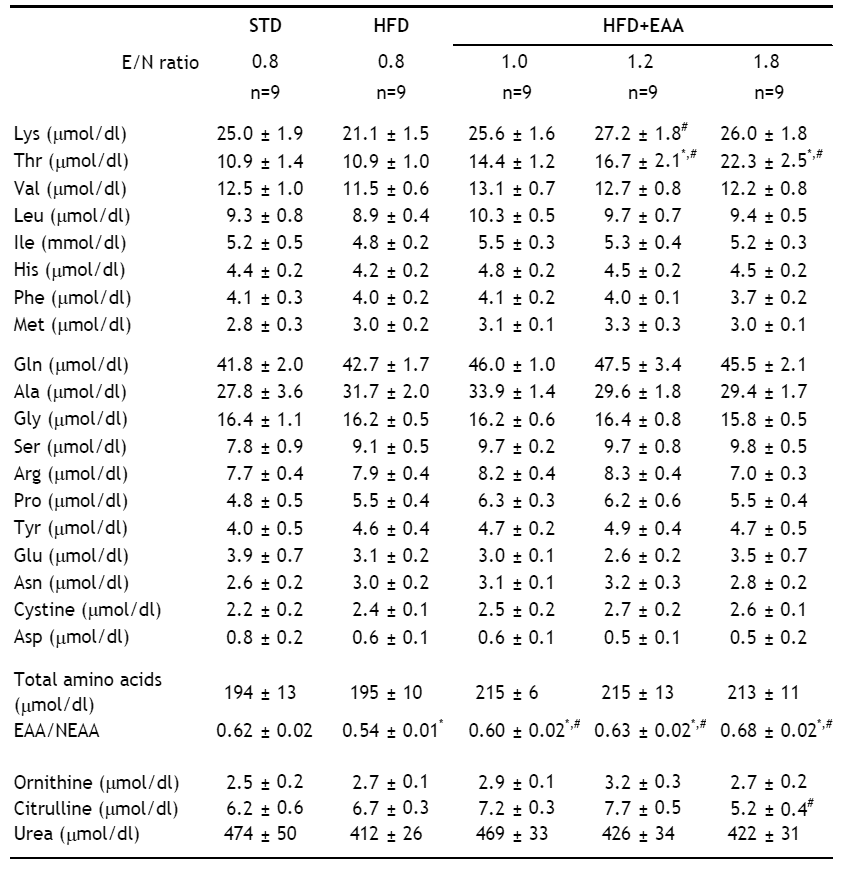

Supplement: Table S4 — Plasma amino acids from c57B6 mice fed HFD for 8 weeks. Data represent mean+/−SEM (n = 6). *, p<0.05 for all high-fat groups compared with STD group; #, p<0.05 for high E/N groups with high fat control. (0.12 MB TIF) [file pone.0012057.s004.tif]

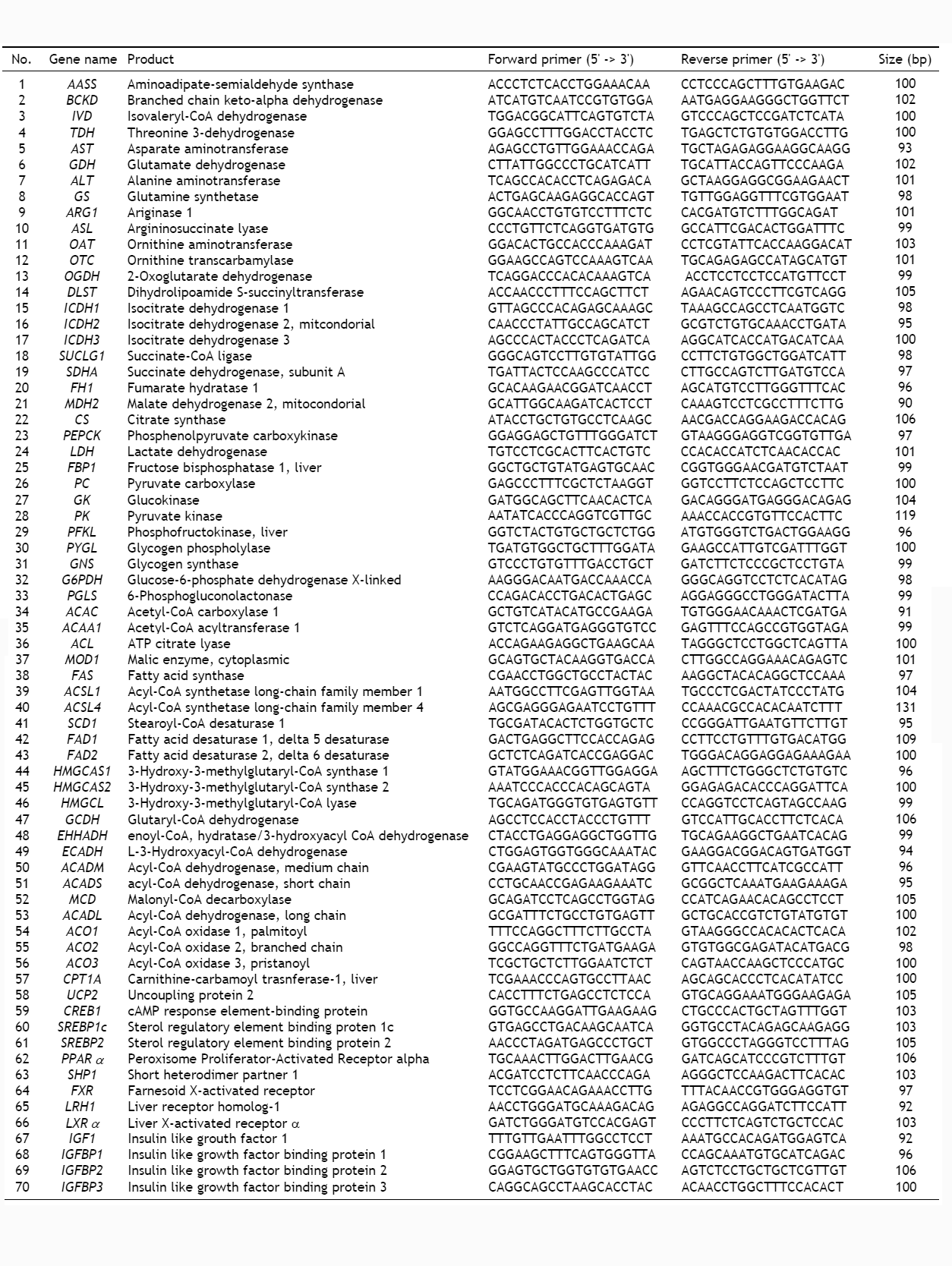

Supplement: Table S5 — Primer sequences used for RT-PCR. (0.47 MB TIF) [file pone.0012057.s005.tif]

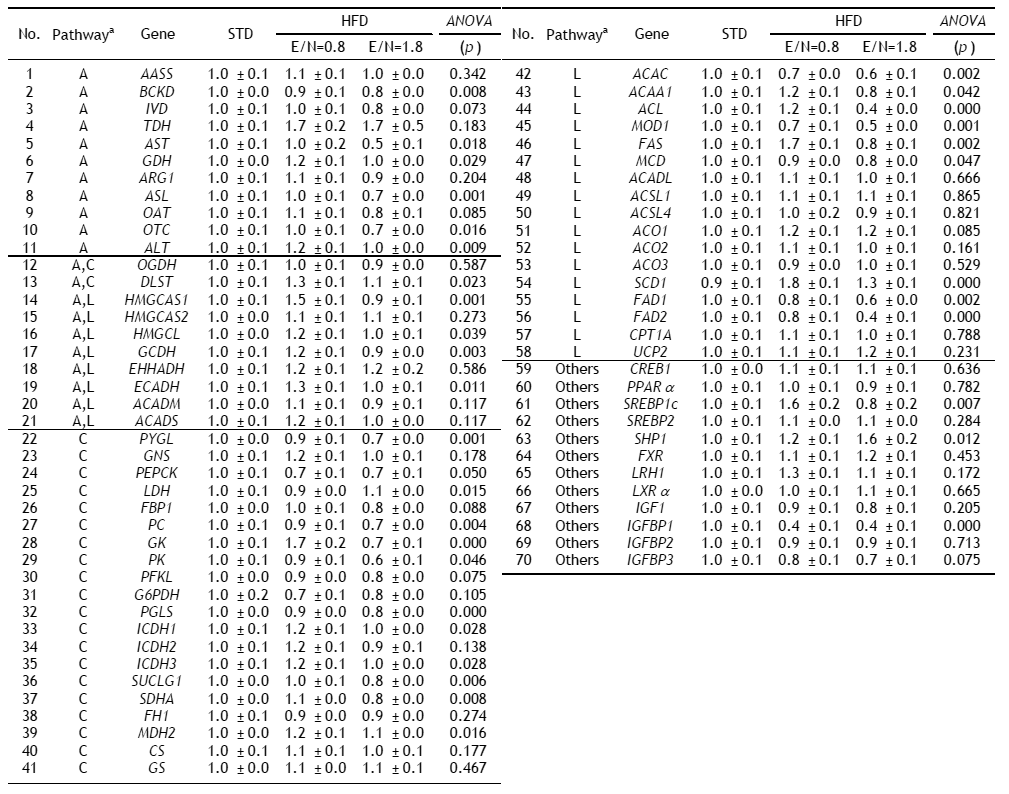

Supplement: Table S6 — Hepatic gene expressions in C57B6 mice. a, Genes are categorized based on KEGG metabolic pathway database. Each letter means the following metabolic pathways: A, amino acid metabolism; L, Lipid metabolism; C, carbohydrate metabolism inculiding glycolysis, gluconeogensis, TCA cycle, glycogen metabolism and pentose phosphate pathway. Data represent mean+/−SEM (n = 9). (0.17 MB TIF) [file pone.0012057.s006.tif]

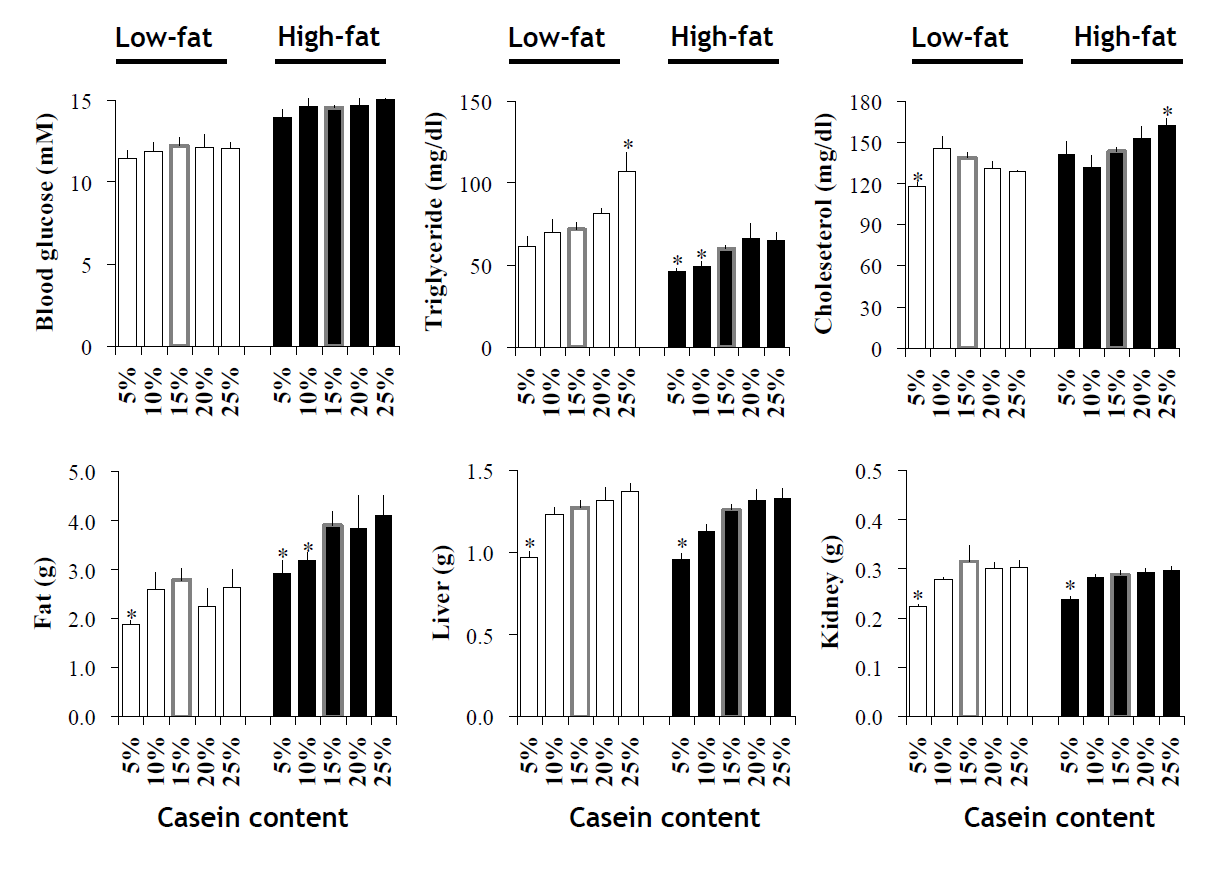

Supplement: Figure S1 — Influences of dietary protein level on metabolic parameters both in low and high fat diet. C57B6 mice were housed with diet containing indicated percent of casein for 8 weeks. Data represent mean+/−SEM (n = 6). *, p<0.05 versus 20% casein group. (0.81 MB TIF) [file pone.0012057.s007.tif]

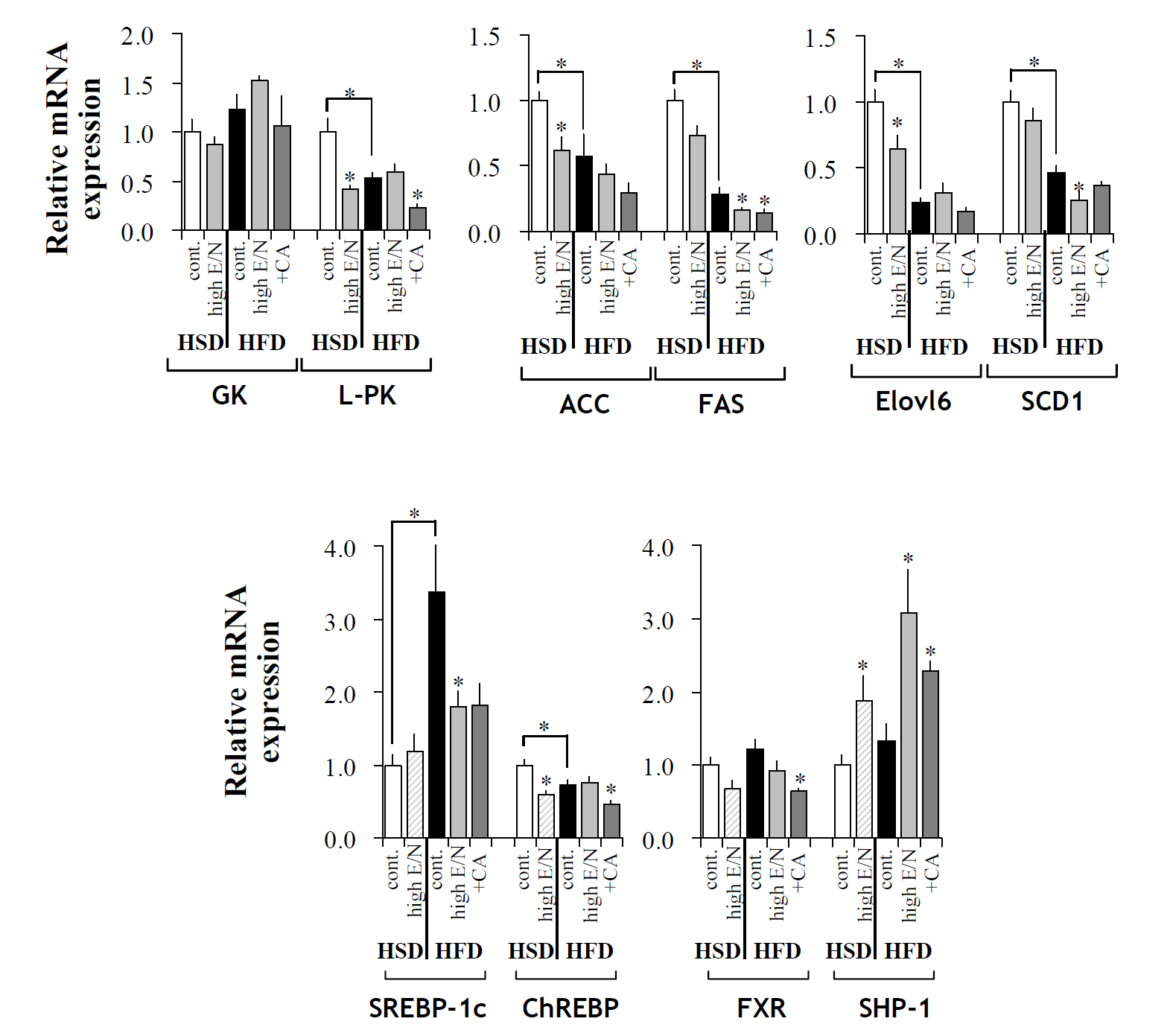

Supplement: Figure S2 — Hepatic expression analysis of lipogenic genes in ob/ob mice. Data represent mean+/−SEM (n = 6). *, p<0.05 for the treatment groups as compared to control or as indicated. (0.44 MB TIF) [file pone.0012057.s008.tif]

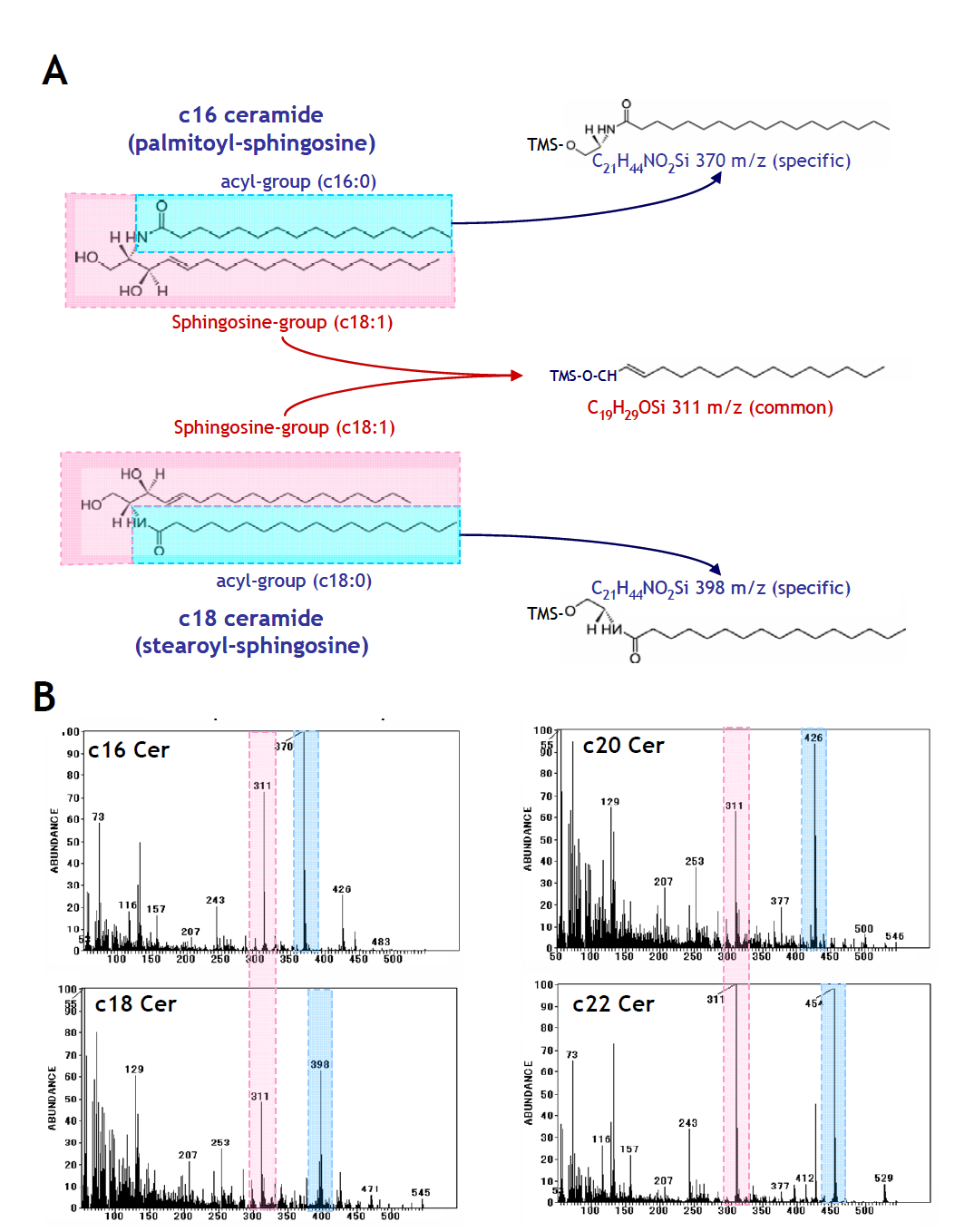

Supplement: Figure S3 — Stable isotopic analysis of ceramide acyl-and sphigonsine groups. Molecular fragments and ions of acyl -and sphingosine groups in TMS-derivatized ceramides (A) and their mass spectra (B). (0.59 MB TIF) [file pone.0012057.s009.tif]
